# Inhibitors of Pathogen Intercellular Signals as Selective Anti-Infective Compounds

**DOI:** 10.1371/journal.ppat.0030126

**Published:** 2007-09-14

**Authors:** Biliana Lesic, François Lépine, Eric Déziel, Jiangwen Zhang, Qunhao Zhang, Katie Padfield, Marie-Hélène Castonguay, Sylvain Milot, Scott Stachel, A. Aria Tzika, Ronald G Tompkins, Laurence G Rahme

**Affiliations:** 1 Department of Surgery, Microbiology and Molecular Genetics, Harvard Medical School, Massachusetts General Hospital, Boston, Massachusetts, United States of America; 2 Department of Microbiology and Molecular Genetics, Harvard Medical School, Boston, Massachusetts, United States of America; 3 Shriners Burns Institute, Boston, Massachusetts, United States of America; 4 INRS-Institut Armand-Frappier, Laval, Québec, Canada; 5 Bauer Center for Genomics Research, Harvard University, Cambridge, Massachusetts, United States of America; Johns Hopkins School of Medicine, United States of America

## Abstract

Long-term antibiotic use generates pan-resistant super pathogens. Anti-infective compounds that selectively disrupt virulence pathways without affecting cell viability may be used to efficiently combat infections caused by these pathogens. A candidate target pathway is quorum sensing (QS), which many bacterial pathogens use to coordinately regulate virulence determinants. The Pseudomonas aeruginosa MvfR-dependent QS regulatory pathway controls the expression of key virulence genes; and is activated via the extracellular signals 4-hydroxy-2-heptylquinoline (HHQ) and 3,4-dihydroxy-2-heptylquinoline (PQS), whose syntheses depend on anthranilic acid (AA), the primary precursor of 4-hydroxy-2-alkylquinolines (HAQs). Here, we identified halogenated AA analogs that specifically inhibited HAQ biosynthesis and disrupted MvfR-dependent gene expression. These compounds restricted P. aeruginosa systemic dissemination and mortality in mice, without perturbing bacterial viability, and inhibited osmoprotection, a widespread bacterial function. These compounds provide a starting point for the design and development of selective anti-infectives that restrict human P. aeruginosa pathogenesis, and possibly other clinically significant pathogens.

## Author Summary

Current treatments of human bacterial infections depend on antibiotics, whose long-term effectiveness is limited as they select for multidrug-resistant pathogens. An alternative approach that is likely to limit the development of bacterial super pathogens is to selectively disrupt bacterial virulence mechanisms without affecting bacterial viability. Quorum sensing (QS), a highly regulated bacterial communication system, is a promising candidate target because it is used by numerous pathogens to stimulate and coordinate the expression of many virulence determinants, and its disruption does not affect bacterial cell viability. We have identified three compounds that efficiently inhibited the synthesis of molecules required for the activation of the human opportunistic pathogen Pseudomonas aeruginosa MvfR-dependent QS regulatory pathway that controls the expression of key virulence genes. We showed that prevention of MvfR pathway activation disrupted MvfR-dependent gene expression, and limited P. aeruginosa infection in mice, without perturbing bacterial viability. In addition, the compounds identified limited the ability of a number of bacterial pathogens to tolerate salt, a widespread bacterial function, and possibly other functions relevant to pathogenesis. These compounds provide a starting point for the design and development of selective anti-infectives that restrict human P. aeruginosa pathogenesis, and possibly other clinically significant pathogens.

## Introduction

Current treatment of human bacterial infections depends on bactericidal and bacteriostatic antibiotics whose long-term effectiveness is limited by the development of drug resistance and can devastate the host commensal microbial community. An alternative approach to combat bacterial pathogens is the use of anti-infective drugs that selectively disrupt pathways that mediate virulence, such as regulation of pathogenesis genes [1]. Compounds that do not disrupt survival or growth should be less likely to generate resistance than traditional antibiotics. Ideally, these reagents should not disrupt bacterial and host metabolism, and should not cause harmful side effects. To date, the development of such drugs has been limited [2–4]. Here, we validated the utility of selective anti-infective compounds to combat infections caused by the opportunistic human pathogen Pseudomonas aeruginosa. This ubiquitous Gram-negative bacterium readily develops antibiotic resistance, and it is a principal agent of deleterious and fatal infections in immunocompromised patients and in pan-antibiotic-resistant outbreaks [5–7]. As such, identification of compounds that selectively disrupt P. aeruginosa pathogenesis should lead to improved clinical treatments of human P. aeruginosa infections.

Bacterial pathogens express certain virulence genes at high cell density. Since this population-dependent regulation controls virulence, but not viability, it is a potential Achilles' heel through which to attack pathogenicity [3,4,8,9]. Many differentiated bacterial behaviors are similarly triggered in response to cell density, and such coordinated intercellular regulation is achieved via quorum sensing (QS), a chemical communication system mediated by small extracellular signal molecules [10]. Signal synthesis is autoinducible, and as such, signal molecule concentration rises as the population density increases until a critical threshold concentration is reached, which then triggers expression of certain sets of genes. However, further studies suggest that the activation of most QS-controlled genes is not solely triggered by the accumulation of signal but also requires additional factors [11,12]. The archetypal QS system uses an acyl homoserine lactone (AHL) intercellular signal that, when the minimum threshold is attained, binds to and activates its cognate LuxR-type transcriptional regulator [13]. This coligand–protein complex then binds as a homodimer to a “lux-box” sequence within the promoters of target loci, including the AHL synthase gene, to activate or repress their transcription [14,15]. Significantly, QS signal molecules occur in infection sites, suggesting that QS inhibition might disrupt virulence [16,17]. Such inhibition could be achieved by interfering with one or more QS components, including signal synthesis, signal regulator binding, or synthase or regulator stability. Although compounds have indeed been identified that inhibit QS in infection sites, to date none have been shown to combat infection effectively. For example, certain AHL derivatives enhance bacterial clearance in lung, and delay death in infected mice, yet fail to reduce overall mortality [18–20]. Similarly, furanones that stimulate LuxR turnover [21] give similar results, yet again do not reduce overall mortality.

Three distinct regulatory pathways have been identified that control QS-dependent expression in *P. aeruginosa.* Two of these systems utilize the LuxR regulatory proteins LasR and RhlR and their cognate AHL autoinducers and synthases [22]. In contrast, the third system utilizes a LysR-type transcriptional regulator (LTTR), MvfR, which is activated by its coligands, 4-hydroxy-2-heptylquinoline (HHQ) and 3,4-dihydroxy-2-heptylquinoline (PQS). Although both HHQ and PQS bind to and activate MvfR, PQS is 100-fold more potent than HHQ [23]. Interestingly, in contrast to the in vitro findings, HHQ is highly produced in vivo, where it is not fully converted into PQS [23]. These coligands are part of a large family of 4-hydroxy-2-alkylquinolines (HAQs) that comprise five distinct congener series, including *N*-oxides, such as 4-hydroxy-2-heptylquinoline *N*-oxide (HQNO) and dihydroxylated derivatives [24–26]. Once MvfR is activated, it binds to a “lys-box” in its target promoters [23,27,28]. MvfR coligand synthesis is also autoinducible [23,29,30]. Production of the coligands is controlled by MvfR, which regulates the production of multiple QS-regulated virulence factors, including pyocyanin, hydrogen cyanide, elastase, and lectins [25,29]. *mvfR* ^−^ mutant cells have greatly reduced pathogenicity in several infection models [31], yet sustain wild-type viability. The attenuated virulence of *lasR^−^* mutant cells is mediated, at least in part, via MvfR [32]. Consequently, selective inhibition of MvfR/HAQ regulation should restrict P. aeruginosa pathogenesis, but not cell viability. Such selective inhibition would also interfere with the ability of P. aeruginosa cells to intercept host stress molecules required to activate P. aeruginosa virulence factors via MvfR and the HAQ signaling molecules PQS and HHQ [33]. Other important bacterial pathogens, such as *Burkholderia* species, also synthesize HAQs [34], which suggests that compounds that selectively inhibit HAQ production could be used as anti-infectives against other pathogens beyond P. aeruginosa.

**Figure 1:**
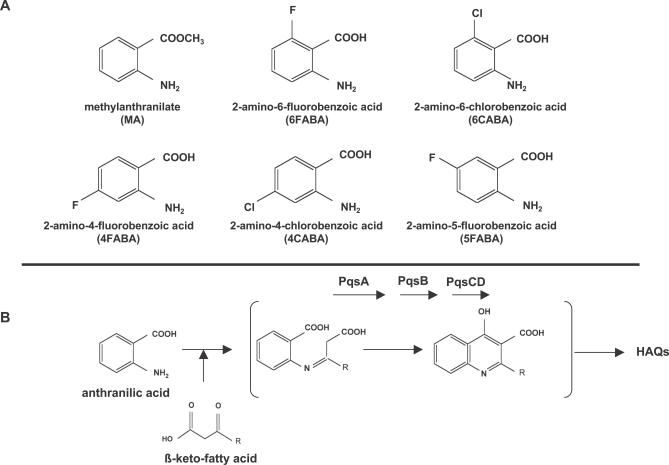
Anthranilic Acid Analogs and HAQ Biosynthetic Pathway (A) Chemical structures of AA, MA, and five halogenated AA analogs. (B) The HAQ biosynthetic pathway and hypothetical steps mediated by the *pqsA-D* gene products.

MvfR coligand synthesis is essential for MvfR activation. This synthesis requires two MvfR-regulated operons: *phnAB* and *pqsA-D*. The operon *phnAB* directs production of anthranilic acid (AA), which, in conjunction with ß-keto fatty acids, is the primary HAQ precursor; while *pqsA-D* directs production of the HAQ congener family [26,35,36]. Also, *pqsH* directs the final HHQ to PQS conversion. An early step in HAQ biosynthesis is hypothesized to be formation of the quinoline 3–4 carbon bond (see Figure 1B) via activation of the AA carbonyl that then reacts with the ß-keto fatty acid methylene. PqsA encodes a predicted coenzyme A ligase, and such ligases can activate aromatic carboxylic acid compounds [37]. According to this scheme, AA analogs that compete with AA for the PqsA protein should inhibit HHQ and PQS synthesis, but not viability, and consequently restrict MvfR activation and bacterial pathogenesis. Here, we identified halogenated AA analogs that specifically inhibited HAQ biosynthesis in both P. aeruginosa and Burkholderia thailandensis cells, disrupted MvfR-dependent gene expression, and reprogrammed the expression of both QS-dependent and QS-independent genes. Significantly, these reagents restricted P. aeruginosa virulence in mice and increased host survival, without perturbing bacterial viability. In addition, they reduced osmoprotection, an environment-related cell response common to many human bacterial pathogens. These compounds provide a starting point for the design and development of selective anti-infective drugs that restrict human–P. aeruginosa pathogenesis, and also possibly other significant pathogens.

## Results

### Identification of Halogenated AA Analogs That Inhibit HHQ and PQS Synthesis

**Figure 2:**
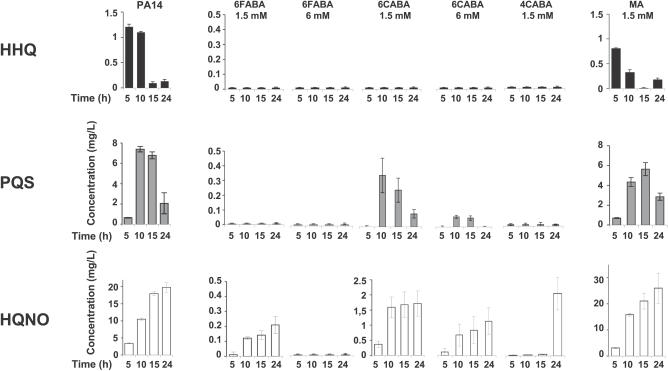
Halogenated AA Analogs Inhibit HAQ Biosynthesis Culture supernatants of PA14 cells grown in LB media minus or plus 1.5 mM or 6 mM 6FABA, 1.5 mM or 6 mM 6CABA, 1.5 mM 4CABA, or 1.5 mM MA, were collected at 5, 10, 15, and 24 h; and HHQ, PQS, and HQNO concentrations (mg/l) were quantified by LC/MS. Data points are the average of triplicate experiments, ± standard deviation. To facilitate presentation, below-detection levels of HAQs are indicated as 0.01.

The AA analog methylanthranilate (MA; Figure 1A) has been previously shown to reduce P. aeruginosa PQS levels [38]. Nevertheless, MA is a poor candidate anti-infective, as 1.5 mM MA only partially reduced the levels of HHQ and PQS, while those of HAQ *N*-oxides were unaffected (Figure 2). Figure 1A presents the five AA analogs tested that carry a fluorine or chlorine atom *ortho* or *para* to the AA carbonyl group: 2-amino-5-fluorobenzoic acid (5FABA), 2-amino-4-fluorobenzoic acid (4FABA), 2-amino-6-fluorobenzoic acid (6FABA), 2-amino-6-chlorobenzoic acid (6CABA), and 2-amino-4-chlorobenzoic acid (4CABA). These analogs were chosen because the electron-withdrawing effects of the halogen atoms should restrict formation of an activated carbonyl such as a CoA ester, and thus synthesis of the second aromatic ring of the HAQ quinoline backbone would be prevented (Figure 1B). However, we cannot exclude the possibility of a steric hindrance effect or the result of an interaction of the halogen with some residues in or near the active site of PqsA (see below). Our results showed that 6FABA, 6CABA, and 4CABA, but not 5FABA and 4FABA, greatly reduced the levels of HHQ, PQS, and HQNO, which are representative congeners of the three principal HAQ series, in PA14 cultures (Figure 2; unpublished data). Furthermore, no chloro-HAQs were detected with 4CABA or 6CABA, and only traces of halogenated *N*-oxides were detected in the presence of 6FABA (unpublished data), indicating that these halogenated AA analogs were not incorporated into HAQs. Also, up to 6 mM 6FABA or 6CABA, and up to 1.5 mM 4CABA did not significantly perturb PA14 growth kinetics (Figure S1). Thus, these concentrations were used in all subsequent experiments, unless noted otherwise.

### The AA Analogs Reprogram P. aeruginosa Gene Expression and Inhibit the MvfR Regulon

The AA analogs 6FABA, 6CABA, and 4CABA markedly reprogram global gene expression in PA14 cells at late exponential growth, when many virulence-related genes are most highly expressed. Comparing the whole-genome transcriptome profile of control cells to the profiles of cells grown in the presence of 6FABA, 6CABA, or 4CABA showed that 354, 618, and 683 genes, respectively, were differentially expressed in response to these compounds (Tables 1 and S1), or 6.2%, 10.8%, and 12% of the 5,684 predicted ORFs assayed by the P. aeruginosa Genechip array. As a high number of genes were identified as differentially expressed, to limit the false positives, we focused on the genes that were affected by all three compounds. Together, these compounds altered the expression of a common set of 205 genes, of which 173 were repressed, and 32 activated (Tables 1 and S1).

**Table 1:**
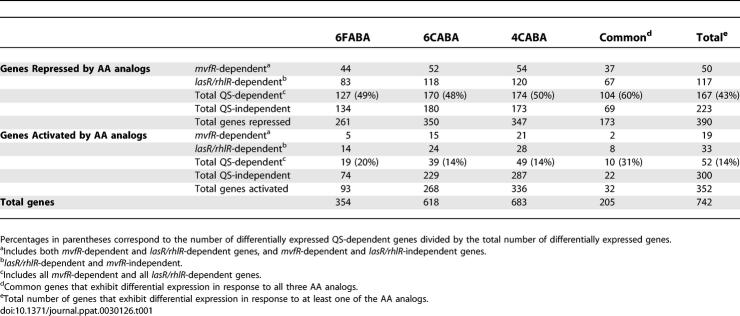
Summary of QS-Dependent and QS-Independent Genes That Are Repressed or Activated in Response to AA Analog Treatment

Inhibition of HHQ and PQS coligand synthesis should prevent MvfR activation, and consequently MvfR-dependent gene regulation. To this end, the AA analogs were found to significantly reprogram MvfR-dependent gene expression, including the suppression of several positively regulated loci that direct HAQ synthesis, or the production of key virulence factors. One hundred forty-four total PA14 genes are MvfR dependent, with 122 positively and 22 negatively regulated [25]. One hundred eleven (78%) of these loci were differentially expressed in response to at least one of the AA analogs (Table S2). Individually, 6FABA, 6CABA, and 4CABA altered the expression of 54, 89, and 102 MvfR-dependent loci, respectively (Table S2). Of the 122 positively regulated MvfR-dependent genes, 56 have known functions, many of which promote P. aeruginosa pathogenicity, including HAQ production [25]. Indeed, all three AA analogs strongly repressed the HAQ biosynthetic operons *pqsA-E* and *phnAB* and the pyocyanin production–mediating operons *phzABCDEFG, phzH, phzM,* and *phzS* (Table S2). In addition, the analogs inhibited several loci that direct the synthesis of additional virulence-related factors, including hydrogen cyanide (*hcnABC*), chitinase (*chiC*), lectins (*lecA* and *lecB*), and elastase (*lasB*) [19,25,31,32,39,40]. Functional assays confirmed that the AA analogs effectively eliminated HAQ biosynthesis (Figure 2) and *pqsA-lacZ* reporter gene expression (Figure S2A), and significantly reduced production of the MvfR-dependent virulence factors, pyocyanin and elastase (Figure S2B and S2C).

QS plays a critical role in the activation of virulence gene expression in many pathogens, and Table 1 shows that the AA analogs strikingly reprogrammed QS-dependent gene expression (also Table S1). Of the 173 common genes that were downregulated by all three analogs, 104 (60%) were under QS control [19,25,41,42], with 37 MvfR-dependent, and 67 solely LasR- and/or RhlR-dependent. Similarly, of the 32 common upregulated loci, 10 were QS dependent (Table 1). This broad perturbation of QS-regulated gene expression further suggested that the AA analogs should potentially limit P. aeruginosa virulence.

Functional classification of the 205 common genes that were differentially expressed in response to all three of the AA analogs revealed different sets of cell activities (unpublished data). Specifically, the repressed genes were overrepresented for activities that include secreted factors, for which the repressed genes strikingly account for almost 20% of all such genes, adaptation and protection functions, such as osmoprotection, and chemotaxis. In contrast, the activated genes were overrepresented for activities that mediate the general cellular machinery and contribute to the cell metabolome. High resolution nuclear magnetic resonance (NMR) analysis of PA14 cells grown minus and plus 4CABA provided functional evidence that the AA analogs stimulate the metabolome, as the assigned resonances for ATP, ADP, and NAD peak resonances were all significantly higher (*p* < 0.05) in the 4CABA-treated cells (Figure S3 and Table S3). Note that 6FABA, 6CABA, and 4CABA upregulated metabolome genes, which further demonstrated they did not inhibit bacterial growth at the concentrations used.

### PqsA as the Target for AA Analog Inhibition of HAQ Production

**Figure 3:**
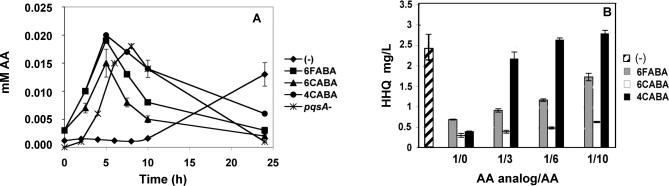
AA Analogs Compete with AA for the PqsA Active Site (A) The AA analogs do not perturb AA accumulation. Intracellular AA concentrations (mM) were determined for PA14 cells grown minus or plus 1.5 mM 6FABA, 6CABA, or 4CABA, and for *pqsA* ^−^ mutant cells. (B) Exogenous AA partially overcomes AA analog inhibition of HHQ synthesis in PA14 cells. Increasing concentrations of AA were added to cultures grown in 0.125 mM 6FABA, 0.75 mM 6CABA, or 0.125 mM 4CABA, which reduced HHQ production by 75% in supernatants of OD_600_ = 4 cell cultures. The competing AA concentrations were 0, 3, 6, and 10 times that of each AA analog.

Through what molecular target do the AA analogs restrict HAQ synthesis? Figure 3A shows that AA accumulated in the cell supernatants of PA14 cells grown in 6FABA, 6CABA, or 4CABA; accordingly, the *antABC* genes, which mediate AA degradation, were strongly upregulated (Table S1). As such, 6FABA, 6CABA, or 4CABA did not restrict HAQ production by inhibiting AA production via PhnAB or another AA synthase. Instead, they most likely shut down HAQ synthesis by competing with AA for the PqsA active site at the start of the HAQ biosynthetic pathway (Figure 1B). To this end, *pqsA^−^* mutant cells also accumulated AA (Figure 3A, and [26]), indicating that the analogs inhibited HAQ biosynthesis activity that utilized AA. Also, exogenous AA partially restored HHQ and PQS production in PA14 cells grown in 6FABA or 4CABA, but not in 6CABA (Figure 3B; unpublished data). If the analogs do directly compete with AA, they should also perturb tryptophan production, since TrpD, the initial enzyme in the tryptophan biosynthesis pathway, utilizes AA as a substrate. Indeed, these compounds inhibited cell proliferation in minimal media, and exogenous tryptophan rescued this defect for the cells grown in 6FABA, or 6CABA, but not 4CABA (Figure S4A). Together, these results suggest that the AA analogs inhibit HAQ production, and at least 6FABA and 6CABA inhibit tryptophan synthesis by respectively competing with AA for the PqsA and TrpD active sites.

### 6FABA, 6CABA, and 4CABA Limit P. aeruginosa Virulence in Mice

**Figure 4:**
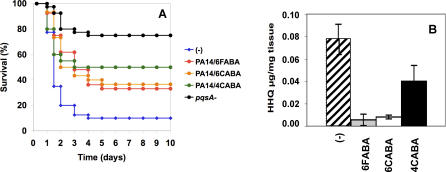
The AA Analogs Promote Survival of B+I Mice to PA14 Pathogenesis, and Restrict In Vivo HHQ Production (A) PA14-infected mice were injected 6 h post-B+I with 6FABA, 6CABA, or 4CABA. Mice infected with *pqsA^−^* cells served as an additional control group. Data were averaged for a minimum of two independent experiments, with *n*(PA14) = 40; *n*(PA14/6FABA) = 36; *n*(PA14/6CABA) = 30; *n*(PA14/4CABA) = 20 and *n*(*pqsA^−^*) = 20. Standard deviations were <20% for the time points between 0 and 4 d, and <12% thereafter. Cox proportional hazards regression showed that mouse mortality due to PA14 infection was significantly reduced by injection with 6FABA (hazard ratio = 0.398, *p* = 5.6e-04), 6CABA (hazard ratio = 0.397, *p* = 1.2e-03), and 4CABA (hazard ratio = 0.325, *P* = 1.8e-03), compared with control saline; *pqsA^−^* virulence was highly attenuated (hazard ratio = 0.118, *p* = 9.1e-06). (B) HHQ levels (*μg*/mg) at 12 h post-B+I for rectus adbominus muscle directly underlying the infection site in untreated B+I mice, and for mice treated 6 h post-B+I with 6FABA, 6CABA, or 4CABA. *n* = 5 for each experimental condition. The *t*-test (*p* = 0.011) and Wilcoxon rank sum test (*p* = 0.03) showed that the difference in HHQ levels between control and 4CABA-treated mice was statistically significant.

The AA analogs should restrict P. aeruginosa virulence, as they prevent the expression of MvfR-dependent virulence genes. Indeed, these compounds limited P. aeruginosa virulence in a thermal injury mice model, where mice were burned and then infected (B+I) with PA14, and subsequently administered with a single intravenous injection of each AA analog at 6 h post-B+I. Figure 4A shows that while only 10% of the uninjected B+I controls survived P. aeruginosa infection, 35%, 37%, and 50% of the mice treated with 6FABA, 6CABA, and 4CABA, respectively, survived infection. In addition, the kinetics of mortality was significantly delayed in the treated mice. As expected, reduced protection was seen for later single injection post-B+I, and essentially no protection was afforded by 24 h (unpublished data).

### 6FABA, 6CABA, and 4CABA Prevent In Vivo HHQ Production, but Not Local Bacterial Proliferation

PA14 cells produce HHQ and PQS in the infection wound site [23]. Figure 4B shows that the AA analogs strongly inhibited this production. The injection of 6FABA or 6CABA at 6 h post-B+I greatly limited in vivo HHQ levels at 12 h post-B+I in rectus abdominus muscle that directly underlies the infection site, versus comparable muscle from untreated B+I mice. This reduction was not due to reduced PA14 proliferation, as the AA analogs did not alter bacterial CFU/mg counts at 12 h post-B+I in the muscle (unpublished data). Although 4CABA was less effective in reducing in vivo HHQ synthesis by 12 h than 6FABA or 6CABA, which could be because the mice received 2.5-fold less 4CABA, it was a better inhibitor of in vitro HAQ synthesis and of MvfR-dependent gene expression than 6FABA or 6CABA. Nevertheless, 4CABA, even with a lower HAQ inhibitory efficacy in vivo, was capable of limiting infection over time similar to 6FABA or 6CABA (Figure 4A).

### HAQ Inhibitors Limit Systemic Bacterial Dissemination in Infected Mice

**Figure 5:**
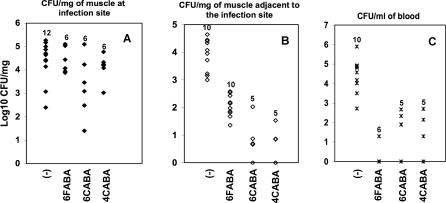
The AA Analogs Restrict Systemic Bacterial Proliferation, but Not Local Proliferation at the Infection Site PA14-infected mice were left untreated (controls) or injected 6 h post-B+I with 6FABA, 6CABA, or 4CABA. The animals were subsequently sacrificed at 22 h post-B+I, and bacteria CFU/mg were determined for (A) muscle tissue that directly underlies the infection site; (B) muscle tissue adjacent to the infection site; and (C) blood. Numbers above dots correspond to the number of mice for each condition. The statistical significance of the CFU/mg differences between the treated and untreated control mice were determined using a Wilcoxon rank sum test. *p*-Values for the difference in adjacent muscle in response to 6FABA, 6CABA, and 4CABA treatment versus no treatment were 0.00001, 0.00066, and 0.00267, respectively; *p*-values for the difference in blood with 6FABA, 6CABA, and 4CABA, treatment versus no treatment were 0.00147, 0.00331, and 0.00331, respectively.

PA14 cells inoculated intradermally into the midline crease of the mouse burn eschar proliferate in the wound, invade the intact underlying rectus abdominus muscle, and then spread via the blood to infect adjacent muscle tissue [43,44]. This systemic dissemination, which is a significant problem in human P. aeruginosa infections, was greatly reduced in mice injected with 6FABA, 6CABA, or 4CABA at 6 h post-B+I, versus uninjected control mice. Figure 5A shows that the bacterial counts in muscle at the infection site were statistically the same for treated and control mice, which further confirmed that the AA analogs did not restrict in vivo bacterial proliferation. Conversely, Figure 5B shows that the PA14 CFU/mg counts in adjacent muscle were 2–3 log units lower in the experimental versus control animals, and Figure 5C shows that the blood bacterial counts were similarly reduced. This inhibition of the systemic dissemination of PA14 cells is likely a major component of the protective in vivo anti-infective efficacy of the AA analogs to limit PA14 pathogenesis. Note that this efficacy is most likely due to inhibition of virulence factor gene expression, versus metabolic perturbation, as isogenic *trpE and trpC* mutant cells proliferated and were as pathogenic as PA14 cells in the B+I mice (unpublished data; Figure S4B), indicating that tryptophan inhibition was non-limiting for in vivo growth and virulence.

### Potential Broad-Spectrum Efficacy: The AA Analogs Reduce HAQ Production and Heighten the Osmosensitivity of Several Bacterial Pathogens

**Figure 6:**
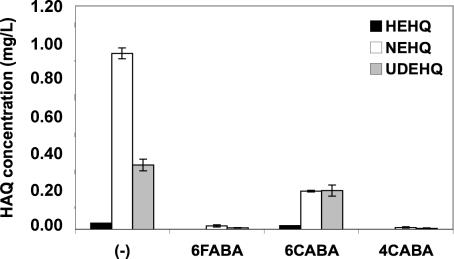
The AA Analogs Inhibit HAQ Production in B. thailandensis Cell Supernatants Mass spectrometry (MS) determination of unsaturated HAQs in OD_600_ = 3 culture supernatants of B. thailandensis cells grown in the absence or presence of 3 mM 6FABA, 6CABA, or 4CABA. Note that while saturated HAQs, including HHQ, occur in B. thailandensis cells, their levels were below the quantification threshold.

Other bacterial pathogens also likely utilize HAQ-based QS signaling and HAQ congeners as virulence mediators. As such, the AA analogs could have broad efficacy as anti-infective reagents against human pathogens beyond P. aeruginosa via their inhibition of HAQ synthesis pathways. To this end, both the highly virulent Gram-negative human pathogen Burkholderia pseudomallei and its close relative B. thailandensis encode functional homologs of the *P. aeruginosa pqsA-E* operon [34] and produce unsaturated HAQs, including HEHQ, NEHQ, and UDEHQ, plus lower levels of saturated HAQs, including HHQ, but not PQS. [34]. Figure 6 shows that 3 mM 6FABA, 6CABA, and 4CABA effectively abolished B. thailandensis HAQ synthesis*.*

**Figure 7:**
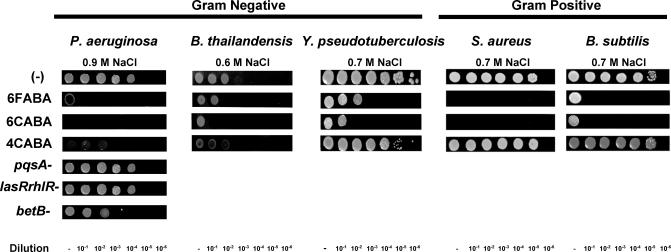
The AA Analogs Increase the Osmotic Sensitivity of Several Bacterial Pathogens Ten times (10×) serial dilutions of the indicated bacterial pathogens were spotted onto high-salt tester plates containing no AA analog, 6 mM 6FABA, 6 mM 6CABA, or 1.5 mM 4CABA. Note that at these concentrations, bacterial growth on regular low-salt LB plates was unaffected.

Virulence genes encode a diversity of activities, several of which permit pathogens to withstand hostile conditions in the host environment, such as high osmolarity. Betaine, a key bacterial osmoprotectant, is synthesized from betaine aldehyde via the *betB* gene product. PA14 cells grown in the presence of 4CABA exhibited significantly higher betaine aldehyde levels versus control cells, as assessed by high resolution NMR (Figure S3 and Table S3), correlating with the lower betaine levels as determined by liquid chromatography/mass spectrometry (LC/MS) and with the reduced *betB* expression in the presence of AA analogs (unpublished data). Figure 7 shows that 6 mM 6FABA, 6 mM 6CABA, and 1.5 mM 4CABA exposure caused PA14 cells to be far less resistant to high salt, versus control cells. This heightened osmosensitivity was not mediated via AA analog inhibition of AHL- or HAQ-mediated QS, as wild-type, *pqsA^−^,* and *lasRrhlR^−^* mutant cells were equally salt resistant, and *betB* expression is QS independent. Instead, this effect was likely due to reduced betaine levels, plus additional QS-independent factors, as *betB^−^* mutant cells were more sensitive to high salt than control cells, but less so than cells grown in the presence of AA analogs. Figure 7 also shows that the AA analogs, especially 6CABA, similarly increased the osmotic sensitivity of the Gram-negative bacteria B. thailandensis and *Yersinia pseudotuberculosis,* and the Gram-positive bacteria Staphylococcus aureus and Bacillus subtilis. Note that 4CABA was highly effective in reducing the salt resistance of *B. thailandensis,* but failed to affect the other tested pathogens. These osmoprotection results further suggest that the AA analogs could have broad-spectrum anti-bacterial activity against pathogens beyond P. aeruginosa.

## Discussion

Combating super pathogens with broad-spectrum resistance may require new anti-infective compounds that selectively interfere with pathways that mediate virulence without affecting cell viability. Here we showed that three halogenated AA analogs, 6FABA, 6CABA, and 4CABA, restricted P. aeruginosa pathogenesis in mice by inhibiting the MvfR-dependent QS system that regulates the expression of key virulence genes. Significantly, these compounds did not perturb cell viability. Four principal results demonstrated the anti-MvfR and anti-pathogenesis efficacy of these AA analogs: 1) they blocked in vivo and in vitro production of HHQ and PQS, and consequently MvfR function; 2) they disrupted the expression of MvfR-dependent virulence genes, including *pqsA-D,* which mediate coligand synthesis; 3) they increased host survival to P. aeruginosa infection; and 4) they restricted systemic bacterial dissemination. These compounds also inhibited HAQ synthesis in B. thailandensis and restricted the production of several non-MvfR-dependent metabolites, including the osmoprotectant betaine, which may promote bacterial survival under harsh host conditions. As such, these compounds could have a multifactorial effect and possibly a broad-spectrum anti-infective activity against several clinically significant human pathogens.

How do the AA analogs disrupt MvfR coligand synthesis? The *pqsA* gene product, which is closely related to coenzyme A ligases that activate aromatic carbonyl compounds [37], is thought to generate the HAQ quinoline backbone from AA and a ß-keto fatty acid. Our hypothesis is that an electron-withdrawing group could inhibit the formation of an activated carbonyl such as a CoA ester, and our results showed that indeed these halogenated AA analogs inhibited HAQ synthesis. We propose that the halogenated analogs compete with AA for the PqsA active site since AA accumulates in PA14 cells grown in the presence of these compounds to levels equivalent to those in *pqsA^−^* mutant cells. Furthermore, exogenous AA reversed 6FABA and 4CABA inhibition of HAQ synthesis, suggesting that they reversibly bind to PqsA. Conversely, AA did not reverse 6CABA inhibition, which suggests that this compound is a non-competitive inhibitor, has much higher PqsA affinity, or has a higher intracellular concentration versus 6FABA or 4CABA. However, unless the PqsA enzyme is purified, it cannot be determined whether AA analogs act on PqsA only through such mechanisms, as it is also possible that the halogen atom could interact with other PqsA residues either inside or close to the active site.

The three compounds also had additional differences to each other, including quantitative and qualitative effects on gene expression, inhibition of osmoprotection, and restoration of tryptophan auxotrophy to cells grown in 6FABA or 6CABA, but not in 4CABA. That 6CABA and 4CABA behave differently—non-competitively in one assay and competitively in another one—in rescuing HHQ synthesis and tryptophan auxotrophy may be due to the fact that PqsA and TrpD enzymes do not have any similarity in protein sequence or domain structure. Therefore, it is possible that these enzymes possess a different AA binding site. The comparison of TrpD and PqsA crystal structures, when they become available, might confirm this. Nevertheless, all three compounds strongly inhibited HHQ and PQS coligand synthesis, and consequently MvfR-dependent gene regulation, and reduced P. aeruginosa pathogenicity. Such effects require that the concentrations of the AA analogs are in the mM range, possibly due to at least either an intrinsic low affinity for the PqsA active site, a reduced ability to enter into the cell, or because they are actively pumped out. If either of the latter two cases occurs, a low concentration of the compounds inside the cells is achieved.

The AA analogs reprogrammed MvfR-dependent and MvfR-independent gene expression. This differential expression response demonstrated the efficacy of the analogs to disrupt MvfR regulon activation and their broader effect on a large number of MvfR-independent loci. Of the 205 genes affected by all three compounds, 173 were repressed, and 32 were activated. That the compounds altered the expression of MvfR-independent loci, indicates they affect additional regulatory pathways, and suggests a possible multifactorial effect.

The mechanism by which AA analogs restrict pathogenicity has not yet been demonstrated. We propose that their anti-infective efficacy is principally due to their inhibition of the MvfR-dependent QS regulatory pathway, though we cannot exclude a multifactorial effect. Interestingly, a single injection of any of the compounds impacted the course of the disease over days. Bacterial numbers and systemic dissemination are major contributing factors to the potential lethality of P. aeruginosa infection, especially in immunocompromised individuals. Indeed, while the AA analogs did not alter bacterial proliferation at the infection site at 12 and 24 h, they significantly lowered bacterial counts in adjacent muscle tissue and prevented systemic spread. These effects, which were not due to reduced tryptophan synthesis and/or viability, likely resulted from inhibition of HAQs, at least up to 12 h, which consequently impacted the expression of the virulence factors dependent on their production, and thus greatly aided the host's ability to clear the infection. As such, MvfR/HAQ inhibition is likely an important component of the anti-infective efficacy of the AA analogs in improving mouse survival to P. aeruginosa infection.

Several plant and human pathogens, including *Pseudomonas, Bordetella, Burkholderia, Ralstonia, Streptomyces, Mycobacteria,* and the Archeae *Sulfolobus,* encode putative PqsA and MvfR homologs, and in some cases, *pqsBCD*-like loci. It would be of interest to determine whether these homologs mediate HAQ production and promote virulence, and if so, whether AA analogs also inhibit them. B. thailandansis and *B. pseudomallei,* a potential biowarfare agent, carry functional *pqsA-D* genes that direct HAQ synthesis [34], and we showed that the AA analogs blocked B. thailandensis HAQ production. These results suggest that the AA analogs may have anti-infective efficacy against bacterial pathogens beyond P. aeruginosa. Furthermore, as virulence functions are often encoded in bacterial pathogens by QS-dependent genes, the effects of AA analogs on MvfR-independent QS-regulated genes also suggests that they may have broad-spectrum activity.

Although QS inhibitors can disrupt QS in vivo, to date, no such compounds have been demonstrated to increase host survival to P. aeruginosa infection [3]. We present here a new class of QS inhibitors that blocked the expression of MvfR-dependent virulence genes. These reagents are the first example of QS pathway inhibitors that restrict P. aeruginosa pathogenesis and improve host survival. Their efficacy confirmed the importance of the MvfR-dependent QS pathway for P. aeruginosa virulence and demonstrated its utility as a therapeutic target for selective anti-infective reagents. Their potential as therapeutic molecules was further supported by the fact that the alternative pathways for AA production, shikimate and tryptophan, are absent in humans. In addition, the ability of AA analogs to restrict production of MvfR-independent compounds, including betaine, could further contribute to their anti-infective activity. Notably, these compounds did not impede bacterial cell viability at the site of infection. Furthermore, the presence of *mvfR* and HAQ synthetic gene homologs in other bacterial pathogens, together with the ability of AA analogs to increase the osmosensitivity of several clinically significant bacteria, suggests that these compounds could be used to treat infections caused by other bacterial pathogens besides P. aeruginosa. It remains to be seen if these compounds cause any significant side effects. Nevertheless, they provide the basis for the design and development of compounds that block the MvfR regulatory pathway. Such reagents should have significant clinical utility in treating acute and chronic P. aeruginosa infections, and possibly other bacterial pathogens.

## Materials and Methods

### Bacterial strains and growth conditions.

The Rif^R^ P. aeruginosa human clinical isolate UCBPP-PA14 [44], and its *pqsA^−^* [26], *lasRrhlR^−^*, (this study), *trpC^−^*, *trpE^−^*, and *betB^−^* [45] isogenic mutant derivatives, were grown at 37 °C on Luria Bertani (LB) agar plates, in LB broth, or in minimal M9 medium plus 2 mM MgSO_4_, 0.4% glucose, and 0.1 mM CaCl_2_. The *lasRrhlR^−^* double mutant was generated by allelic exchange using the previously constructed single *rhlR*::Tc PA14 [25] and *lasR*::Gm PA14 mutants [26]. B. thailandensis E264, S. aureus 8325, and *B. subtilis* QPB467 were grown at 37 °C on LB agar or in LB broth, and Y. pseudotuberculosis IP32953 was grown at 30 °C on LB agar or in LB broth. One hundred *μ*g ml^−1^ rifampicin, 300 *μ*g ml^−1^ carbenicillin, 30 *μ*g ml^−1^ gentamycin, tetracycline (100 μg/ml), 1 mM tryptophan, and different concentrations of AA were used as required.

### AA analogs.

For bacterial assays, a fresh solution of each compound (Sigma-Aldrich, http://www.sigmaaldrich.com/) was prepared in culture medium. For mice injections, fresh 20-mM solutions of 6FABA or 6CABA were prepared in 0.9% NaCl, dissolved at 50 °C for 30 min, and filtered through 0.22-μm filters. For 4CABA, a 20-mM solution in 50% ethanol was first prepared, as 4CABA has a maximal solubility of 3 mM in 0.9% NaCl.

### PA14 growth.

Overnight PA14 cultures were grown in LB minus or plus AA analog and diluted the following day in fresh media minus or plus compound. Bacterial growth kinetics were determined by measuring OD_600_. The maximal concentrations that do not restrict PA14 growth in LB were found to be 6 mM 6FABA, 6 mM 6CABA, and 1.5 mM 4CABA. These concentrations were used in all subsequent experiments, unless otherwise noted.

### P*pqsA-lacZ* expression.

Overnight cultures of PA14 and *pqsA^−^* cells harboring pGX5, which carries the P*pqsA*-*lacZ* reporter gene [27], were diluted to OD_600_ 0.05; and β-galactosidase activity, expressed as Miller Units [46], and OD_600_ were measured at selected time points. Assays were performed in triplicate.

### Pyocyanin and elastase quantification.

Pyocyanin concentration (μg/ml) was determined by measuring OD_520_ [47,48]. Elastase activity was determined by measuring the OD_495_ of 1 ml of OD_600_ = 4 culture supernatant mixed with 10 mg of elastin congo red (Sigma-Aldrich), following incubation at 37 °C for 3 h with agitation and centrifugation.

### RNA isolation and transcriptome analyses.

PA14 cells were grown in 5 ml of LB at 37 °C with agitation minus and plus 6 mM 6FABA, 6 mM 6CABA, or 1.5 mM 4CABA. Triplicate samples of two independent cultures for each AA analog were harvested at OD_600_ = 2.5, and total RNA was purified using the RNAeasy spin column (Qiagen, http://www.qiagen.com/), and assayed using the GeneChip P. aeruginosa Genome Array (Affymetrix, http://www.affymetrix.com/).

### GeneChip expression.

Affymetrix DAT files were processed using the Affymetrix Gene Chip Operating System (GCOS) to create .*cel* files*.* The raw intensity .*cel* files from the 12 chips, three replicates each for four different conditions, were normalized by robust multi-chip analysis (RMA) (Bioconductor release 1.7) with PM-only models. Array quality control metrics generated by Affymetrix Microarray Suite 5.0 were used to assess hybridization quality. Normalized expression values were analyzed with SAM (Significance Analysis of Microarray) [49] using the permuted unpaired two-class test. The control group consisted of sample replicates in the absence of AA analogs. Each of the three experimental groups consisted of the AA analog sample replicates. Genes whose transcript levels exhibited an up or down absolute fold change >2, and *q* value <6% in response to all three analogs versus control were further analyzed. Functional annotation for the differentially regulated genes is from http://v2.pseudomonas.com/.

### Statistics.

The likelihood of overrepresentation of functional categories in the upregulated or downregulated genes relative to the background of all array genes was calculated using Fisher's exact test. The statistical significance differences of PA14 CFU/mg between experimental and control groups were calculated using the Wilcoxon rank sum test. The statistical significance differences in the metabolome of PA14 cells grown in absence or presence of 4CABA were calculated using the independent samples *t*-test (equal variances, two-tailed; α = 0.05). Significance of survival kinetics was calculated using Kaplan–Meier analysis with assessment of statistical significance using the Mantel–Cox log-rank test and Cox model. The statistical significance difference in HHQ levels in vivo between control and treated mice was calculated using the *t*-test and Wilcoxon rank sum test.

### LC/MS.

The quantification of HAQs in bacterial culture supernatants and in infected mouse tissue was performed as described [23,50]. The HAQs were separated on a C18 reverse-phase column connected to a triple quadrupole mass spectrometer, using a water/acetonitrile gradient [50]. Positive electrospray in MRM mode with 2 × 10^−3^ mTorr argon and 30 V as the collision gas and energy was employed to quantify HAQs, using the ion transitions HHQ 244>159, HHQ-D4 248>163, HQNO 260>159, PQS 260>175, and PQS-D4 264>179. B. thailandensis HAQs were assessed as above. The pseudomolecular ions of each compound were monitored in full scan mode, using the unsaturated PA14 HAQ response factors. 

### High-resolution magic angle spinning proton nuclear magnetic resonance.

PA14 cells were grown in triplicate minus or plus 1.5 mM 4CABA to OD_600_ = 3. The culture samples were centrifuged and the pellets were washed with ice cold PBS, resuspended in 100 μl of 75% prewarmed ethanol, and sonicated. Four hundred μl of 75% prewarmed ethanol was added and the samples were incubated at 100 °C for 4 min. Bacterial debris were removed by centrifugation, and the supernatants were dried in a SpeedVac (Thermo Scientific, http://www.thermo.com/) and dissolved in 100 mM phosphate prepared with D_2_O. An aliquot of 10–20 μl of each sample was pipetted into a 4-mM rotor with a spherical insert, and 10–20 μl D_2_O containing 50 mM TSP (trimethylsilyl propionic-2,2,3,3-d_4_ acid, M_w_ = 172, d = 0 ppm) was added to provide the deuterium lock and external chemical shift references, respectively. High-resolution magic angle spinning proton nuclear magnetic resonance (HRMAS ^1^H NMR) was performed on a Bruker BioSpin (http://www.bruker-biospin.com/) Avance NMR spectrometer (proton frequency at 600.13 MHz, 89-mm vertical bore) using a 4-mM triple resonance (^1^H, ^13^C, ^2^H) HRMAS probe (Bruker). Temperature was maintained at 4 °C by a BTO-2000 unit in combination with a MAS pneumatic unit (Bruker). The MAS speed was stabilized at 4.0 ± 0.001 kHz by a MAS speed controller. One dimensional ^1^H NMR spectra were acquired for all the samples using a rotor-synchronized Carr-Purcell-Meiboom-Gill (CPMG) spin echo pulse sequence, (90°-(t-180°-t)_n_-acquisition), which works as a T_2_ filter to remove the spectral broadening. The inter-pulse delay (t) was synchronized to the MAS speed of 250 μs. The number of transients was 256 with 32,768 (32 k) data points. A line-broadening apodization function of 1.0 Hz was applied to all HRMAS ^1^H FIDs prior to Fourier transformation. Metabolite chemical shifts were according to Bundy et al. [51] and Chauton et al. [52].

### Mouse mortality.

The animal protocol was approved by the Massachusetts General Hospital Institutional Animal Care and Use Committee. A thermal injury mouse model [43] was used as described previously [44] to assess bacterial pathogenicity in 6-wk-old CD1 mice (Charles River Laboratories, http://www.criver.com/). Following mouse anesthetization, a full-thickness thermal burn injury involving 5%–8% of the body surface area was produced on the dermis of the shaved abdomen, and an inoculum of 5 × 10^5^ PA14 cells was injected intradermally into the burn eschar. To assess the anti-infective efficacy of the AA analogs to limit P. aeruginosa virulence, B+I mice received a single IV injection of 100 μl of 20 mM 6FABA (12 μg/g body weight), 100 μl of 20 mM 6CABA (13.6 μg/g), or 40 μl of 20 mM 4CABA (5.4 μg/g). Note that these compounds were injected at 6 h post-B+I because HHQ and PQS were not detected in vivo at that time point. Mice received a lower dose of 4CABA, as they appeared disoriented when a higher amount was used. Such an effect may be due to the fact that 4CABA was dissolved in EtOH instead of saline, which was used for both 6FABA and 6CABA. Mice survival was subsequently assessed over 7 d. Experiments were repeated at least in duplicate. Mice infected with *pqsA^−^* mutant cells, whose virulence was attenuated in B+I mice versus PA14 cells [25], served as additional controls. Note that injection of each of the compounds tested is nontoxic.

### P. aeruginosa local and systemic proliferation in mice.

Four sets of B+I mice were burned and infected with PA14. Three of these sets were injected 6 h post-infection with 6FABA, 6CABA, or 4CABA, with the uninjected mice serving as controls. Five to 12 mice from each set were sacrificed at 12 or 22 h post-B+I, and blood samples and tissue biopsies of rectus abdominus muscle directly underlying the infection site, or adjacent to this site, were collected for quantification of bacterial CFU/mg tissue. Blood samples of 50 *μ*l or 5 *μ*l were immediately plated on agar, muscle tissue was homogenized in 2 ml of PBS, and serial dilutions were plated on LB Rif plates. Following 24 h incubation at 37 °C, PA14 CFU were determined. Note that although bacteria can directly invade muscle that closely underlies the infection site, they require systemic delivery via the blood to infect adjacent muscle.

### Osmotic stress.

Starting from OD_600_ = 3 bacterial cultures, a series of six 10-fold dilutions were spotted onto high-salt LB agar tester plates minus AA analog, or plus 6 mM 6FABA, 6 mM 6CABA, or 1.5 mM 4CABA. Concentrations of 0.9 M, 0.6 M, and 0.7 M NaCl were respectively used to osmotically stress *P. aeruginosa; B. thailandensis;* and *S. aureus, B. subtilis,* and *Y. pseudotuberculosis* cells. Plates were read after overnight incubation. Note that the compounds do not perturb bacterial growth under the low osmotic conditions of regular LB plates.

## Supporting Information

Figure S1 Growth Kinetics of PA14 in Response to 6FABA, 6CABA, 4CABA, or MA (89 KB PDF)

Figure S2 The AA Analogs 6FABA, 6CABA, and 4CABA Inhibit (A) Transcription from *pqsA-E* Promoter, (B) Pyocyanin, and (C) Elastase Production Data are average of triplicate experiments ± SD. Three independent experiments were performed.
(171 KB PDF)

Figure S3 The 600-MHz HRMAS ^1^H NMR Spectra of PA14 Cell Samples Grown in the Presence or Absence of 1.5 mM 4CABA The ^1^H chemical shift assignments are labeled as 0, TSP; 1, lipids/macromolecules; 2, lactate; 3, alanine; 4, acetate; 5, glutamate; 6, glutamine; 7, lysine; 8, histidine; 9, choline; 10, betaine aldehyde; 11, glycine; 12, amino acid; 13, betaine aldehyde; 14, residual water; 15, C/UXP; 16, ADP+ATP; 17, C/UXP; 18, NAD; 19, ADP+ATP; and 20–23, NAD.
(56 KB PDF)

Figure S4 Tryptophan Deficiency Does Not Contribute to the Analog-Mediated Decreased *P. aeruginosa* Virulence in Mice (A) Growth kinetics of PA14 in minimal medium in the presence or absence of 6 mM 6FABA, 6 mM 6CABA, or 1.5 mM 4CABA plus or minus 1 mM tryptophan.
(B) Virulence of PA14 wild-type, and *trpE^−^*, *and trpC^−^* mutants in the B+I model.
(165 KB PDF)

Table S1 Differential Expression Ratios of Genes that Are Positively and Negatively Regulated in Response to 6FABA, 6CABA, and 4CABA (484 KB DOC)

Table S2 Differential Expression Levels of MvfR Positively and Negatively Regulated Genes in Response to 6FABA, 6CABA, or 4CABA (297 KB DOC)

Table S3 Levels of Prominent ^1^H HRMAS MR Spectra Metabolite Peaks in PA14 Cells Minus or Plus 4CABA Treatment (50 KB DOC)

## Abbreviations

AA: anthranilic acid
AHL: acyl homoserine lactone
B+I: burn and infection
HAQ: 4-hydroxy-2-alkylquinoline
HHQ: 4-hydroxy-2-heptylquinoline
HQNO: 4-hydroxy-2-heptylquinoline *N*-oxide
HRMAS ^1^H: high-resolution magic angle spinning proton
LC/MS: liquid chromatography/mass spectrometry
NMR: nuclear magnetic resonance
MA: methylanthranilate
PQS: 3,4-dihydroxy-2-heptylquinoline
QS: quorum sensing
