# Supplementary figures and images for: Inhibitors of Pathogen Intercellular Signals as Selective Anti-Infective Compounds
Source: PLoS Pathog. 2007 Sep 14;3(9):e126. doi: 10.1371/journal.ppat.0030126 (PMC2323289; doi:10.1371/journal.ppat.0030126)

**Figure S1: Growth kinetics of PA14 in response to 6FABA, 6CABA, 4CABA, or methylantranilate (MA).**

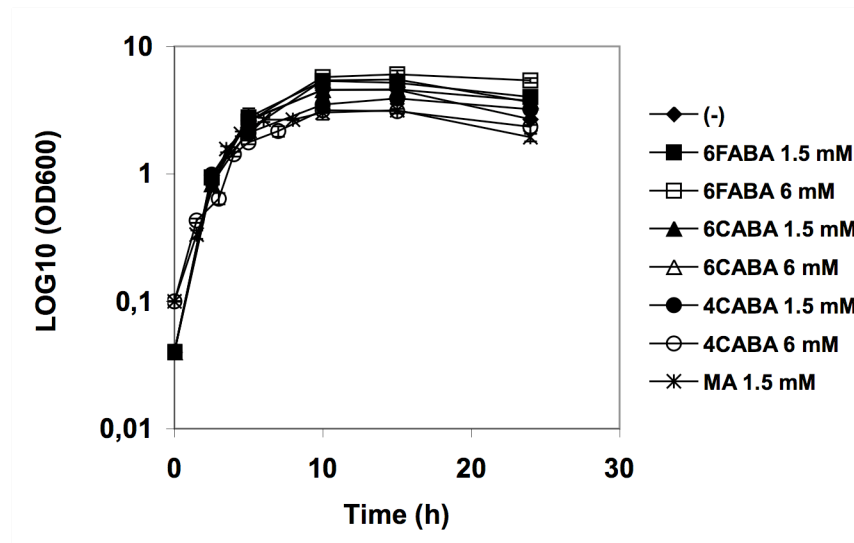

Supplement: Figure S1 — (89 KB PDF) [file ppat.0030126.sg001.pdf]
