# Supplementary material for: Inhibitors of Pathogen Intercellular Signals as Selective Anti-Infective Compounds
Source: PLoS Pathog. 2007 Sep 14;3(9):e126. doi: 10.1371/journal.ppat.0030126 (PMC2323289; doi:10.1371/journal.ppat.0030126)

Figure S2: The AA analogs 6FABA, 6CABA, and 4CABA inhibit A) transcription from *pqsA-E* promoter, **B)** pyocyanin, and **C)** elastase production.

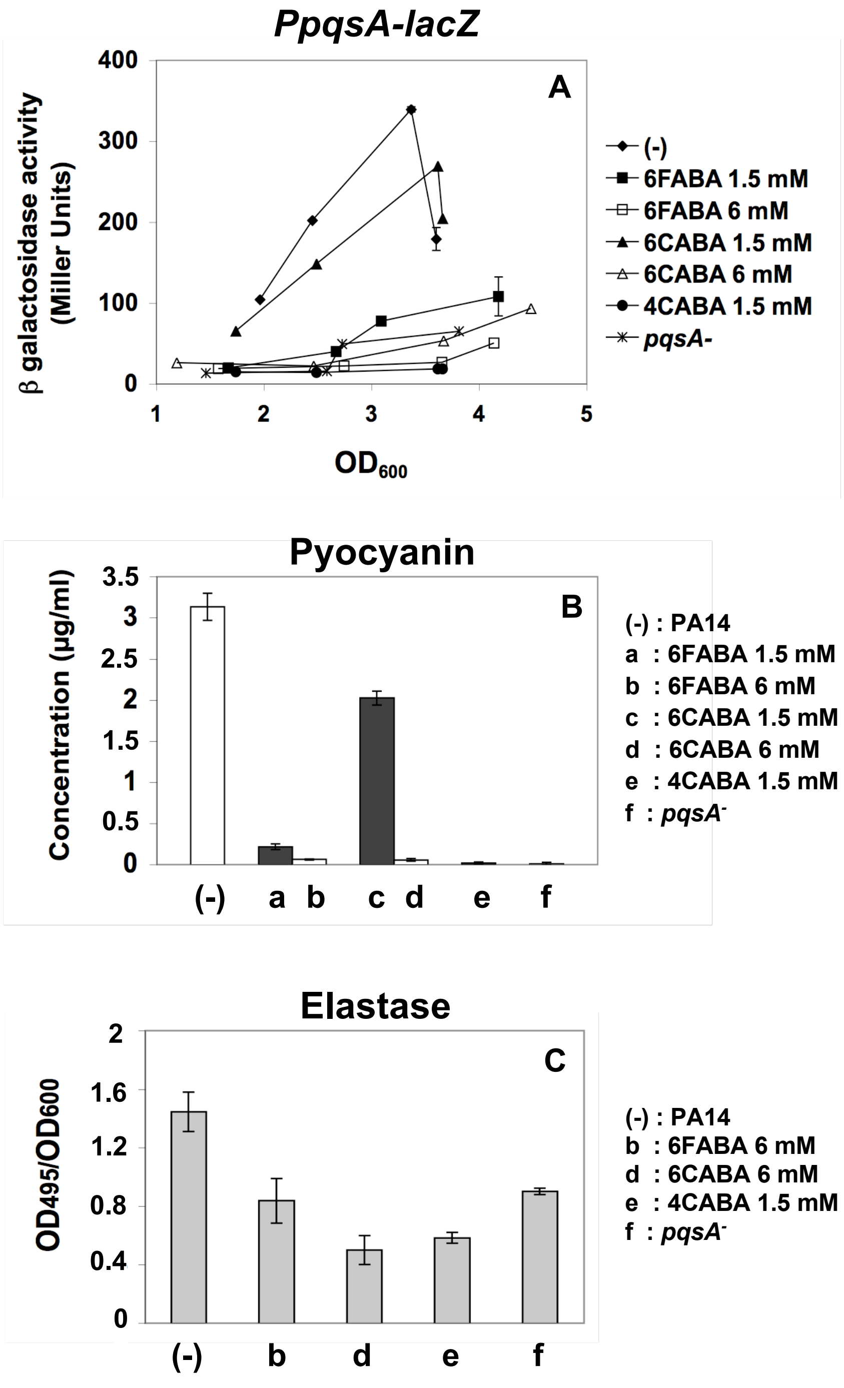

Supplement: Figure S2 — Data are average of triplicate experiments ± SD. Three independent experiments were performed. (171 KB PDF) [file ppat.0030126.sg002.pdf]
