# Supplementary material for: Inhibitors of Pathogen Intercellular Signals as Selective Anti-Infective Compounds
Source: PLoS Pathog. 2007 Sep 14;3(9):e126. doi: 10.1371/journal.ppat.0030126 (PMC2323289; doi:10.1371/journal.ppat.0030126)

**Figure S3: The 600MHz high-resolution Magic Angle Spinning proton (HRMAS $^{1}H$) nuclear magnetic resonance (NMR) spectra of PA14 cell samples grown in the presence or absence of 1.5 mM 4CABA.**

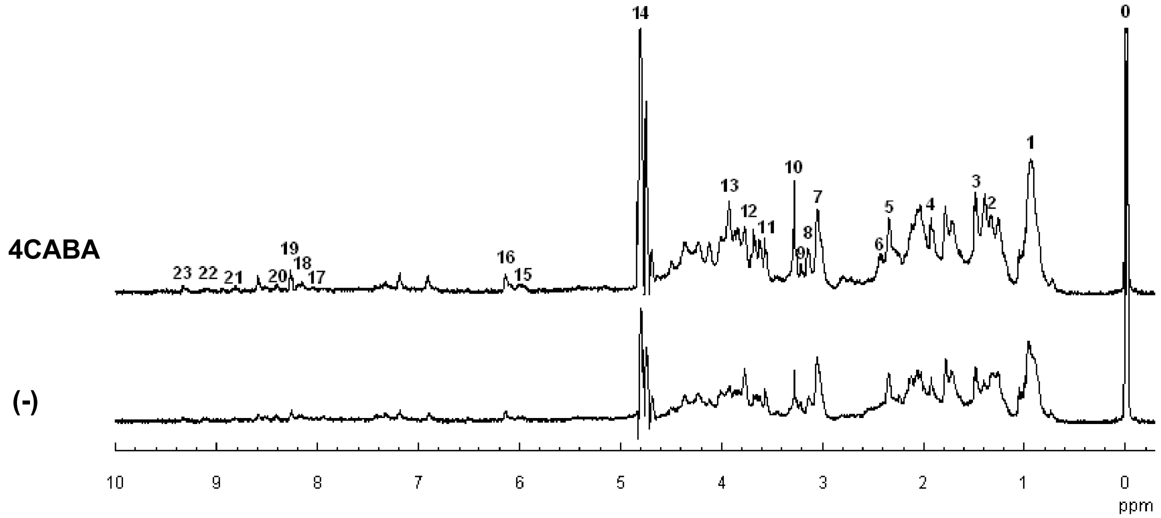

Supplement: Figure S3 — The 1H chemical shift assignments are labeled as 0, TSP; 1, lipids/macromolecules; 2, lactate; 3, alanine; 4, acetate; 5, glutamate; 6, glutamine; 7, lysine; 8, histidine; 9, choline; 10, betaine aldehyde; 11, glycine; 12, amino acid; 13, betaine aldehyde; 14, residual water; 15, C/UXP; 16, ADP+ATP; 17, C/UXP; 18, NAD; 19, ADP+ATP; and 20–23, NAD. (56 KB PDF) [file ppat.0030126.sg003.pdf]
