# Supplementary material for: Inhibitors of Pathogen Intercellular Signals as Selective Anti-Infective Compounds
Source: PLoS Pathog. 2007 Sep 14;3(9):e126. doi: 10.1371/journal.ppat.0030126 (PMC2323289; doi:10.1371/journal.ppat.0030126)

Figure S4: A) Growth kinetics of PA14 in minimal medium in the presence or absence of 6 mM 6FABA, 6 mM 6CABA, or 1.5 mM 4CABA plus or minus 1 mM tryptophan. B) Virulence of PA14 wild type, and *trpE*⁻ *and trpC*⁻ mutants in the burn and infection (B+I) model.

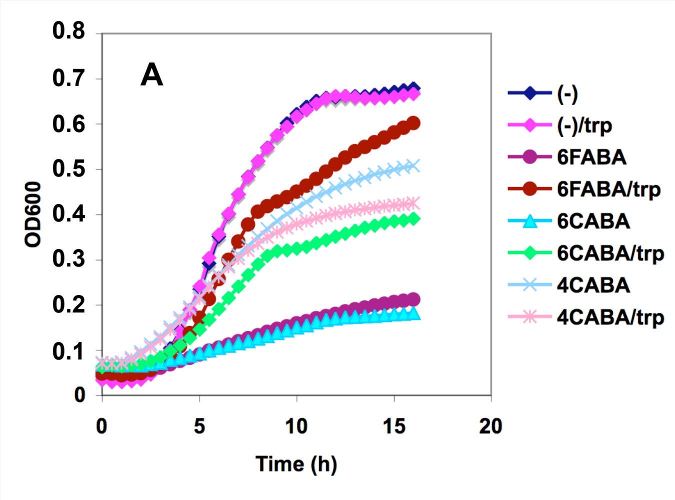

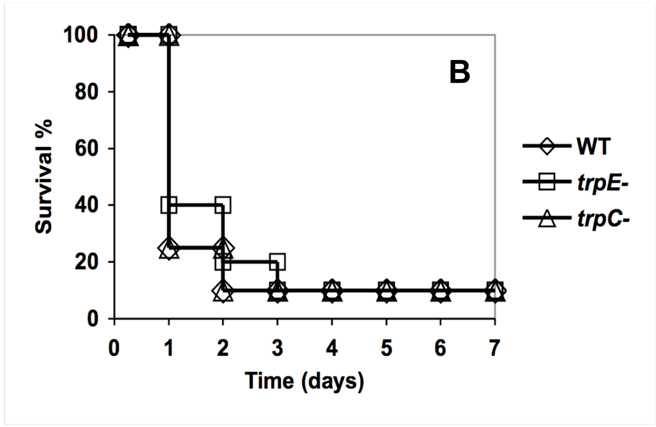

Supplement: Figure S4 — (A) Growth kinetics of PA14 in minimal medium in the presence or absence of 6 mM 6FABA, 6 mM 6CABA, or 1.5 mM 4CABA plus or minus 1 mM tryptophan. (B) Virulence of PA14 wild-type, and trpE−, and trpC− mutants in the B+I model. (165 KB PDF) [file ppat.0030126.sg004.pdf]
