# Supplementary material for: Inhibitors of Pathogen Intercellular Signals as Selective Anti-Infective Compounds
Source: PLoS Pathog. 2007 Sep 14;3(9):e126. doi: 10.1371/journal.ppat.0030126 (PMC2323289; doi:10.1371/journal.ppat.0030126)
Supplement: Table S1 — (484 KB DOC) [file ppat.0030126.st001.doc]

**Table S1: Differential expression ratios of genes that are positively and negatively regulated in response to 6FABA, 6CABA, and 4CABA**

| **Identifier** | **QS regulation** | | **6FABA** | **6FABA** | **6CABA** | **4CABA** | **4CABA** | **4CABA** | **Gene features** | **Functional classification** |
| --- | --- | --- | --- | --- | --- | --- | --- | --- | --- | --- |
|  |  |  | **Fold change** | **q-value (%)** | **Fold change** | **q-value (%)** | **Fold change** | **q-value (%)** |  |  |
| **Genes supressed by AA analogs** | | |  |  |  |  |  |  |  | |
|  |  |  |  |  |  |  |  |  |  |  |
| PA0051_phzH | ***mvfR*** |  | -5,02 | 0,00 | -5,23 | 0,00 | -6,01 | 0,00 | potential phenazine-modifying enzyme | Putative enzymes |
| PA0052 |  | ***lasR+rhlR*** | -2,12 | 5,15 | -4,65 | 0,00 | -3,35 | 0,00 | hypothetical protein | Hypothetical, unclassified, unknown |
| PA0059_osmC |  | ***lasR+rhlR*** | -2,24 | 0,76 | -2,48 | 0,75 | -2,31 | 0,00 | osmotically inducible protein OsmC | Adaptation Protection |
| PA0122 | ***mvfR*** | ***lasR+rhlR*** | -3,40 | 0,00 | -3,42 | 0,00 | -2,74 | 0,00 | conserved hypothetical protein | Hypothetical, unclassified, unknown |
| PA0129_gabP |  |  | -3,14 | 2,77 | -3,28 | 0,00 | -15,56 | 0,00 | gamma-aminobutyrate permease | Transport of small molecules |
| PA0130 |  |  | -4,02 | 0,94 | -3,23 | 0,00 | -14,80 | 0,00 | probable aldehyde dehydrogenase | Putative enzymes |
| PA0131 |  |  | -3,91 | 0,94 | -2,33 | 0,28 | -14,75 | 0,00 | hypothetical protein | Hypothetical, unclassified, unknown |
| PA0132 |  | ***lasR+rhlR*** | -5,61 | 0,00 | -3,50 | 0,00 | -21,45 | 0,00 | beta-alanine--pyruvate transaminase | Amino acid biosynthesis and metabolism |
| PA0140_ahpF |  |  | -2,85 | 0,94 | -3,26 | 0,00 | -2,64 | 0,00 | alkyl hydroperoxide reductase subunit F | Adaptation Protection |
| PA0173 |  |  | -2,68 | 0,00 | -2,58 | 0,00 | -2,29 | 0,00 | probable methylesterase | Adaptation Protection Chemotaxis |
| PA0175 |  | ***lasR+rhlR*** | -1,69 | 4,66 | -2,07 | 0,00 | -1,88 | 0,00 | probable chemotaxis protein methyltransferase | Transcriptional regulators |
| PA0176 |  | ***lasR+rhlR*** | -2,26 | 0,76 | -3,07 | 0,00 | -2,47 | 0,00 | probable chemotaxis transducer | Adaptation Protection Chemotaxis |
| PA0250 |  |  | -4,83 | 0,00 | -3,64 | 0,13 | -2,90 | 0,00 | conserved hypothetical protein | Hypothetical, unclassified, unknown |
| PA0269 |  |  | -3,55 | 0,00 | -3,23 | 0,00 | -3,33 | 0,00 | conserved hypothetical protein | Hypothetical, unclassified, unknown |
| PA0270 |  |  | -3,80 | 0,00 | -3,84 | 0,00 | -4,31 | 0,00 | hypothetical protein | Hypothetical, unclassified, unknown |
| PA0271 |  |  | -3,71 | 0,00 | -3,89 | 0,00 | -4,18 | 0,00 | hypothetical protein | Hypothetical, unclassified, unknown |
| PA0315 |  |  | -3,45 | 0,00 | -2,82 | 0,13 | -2,42 | 0,00 | hypothetical protein | Hypothetical, unclassified, unknown |
| PA0355_pfpI | ***mvfR*** | ***lasR+rhlR*** | -2,20 | 3,89 | -2,09 | 0,46 | -2,36 | 0,00 | protease PfpI | Translation, post-translational modification, degradation |
| PA0430_metF |  |  | -3,84 | 3,44 | -2,69 | 0,13 | -2,78 | 0,00 | 5,10-methylenetetrahydrofolate reductase | Amino acid biosynthesis and metabolism |
| PA0431 |  |  | -4,57 | 0,00 | -4,20 | 0,00 | -3,70 | 0,00 | hypothetical protein | Hypothetical, unclassified, unknown |
| PA0432_sahH |  |  | -4,45 | 0,76 | -3,53 | 0,00 | -2,42 | 0,00 | S-adenosyl-L-homocysteine hydrolase | Amino acid biosynthesis and metabolism |
| PA0546_metK |  |  | -4,52 | 0,00 | -3,21 | 0,00 | -3,97 | 0,00 | methionine adenosyltransferase | Amino acid biosynthesis and metabolism |
| PA0572 |  | ***lasR+rhlR*** | -2,79 | 0,94 | -2,73 | 0,21 | -2,77 | 0,00 | hypothetical protein | Hypothetical, unclassified, unknown |
| PA0588 |  | ***lasR+rhlR*** | -2,26 | 0,94 | -2,70 | 0,00 | -2,18 | 0,00 | conserved hypothetical protein | Hypothetical, unclassified, unknown |
| PA0704 |  |  | -2,31 | 0,00 | -3,21 | 0,00 | -2,33 | 0,00 | probable amidase | Putative enzymes |
| PA0803 |  |  | -2,25 | 0,00 | -2,48 | 0,00 | -2,04 | 0,00 | hypothetical protein | Hypothetical, unclassified, unknown |
| PA0848 |  |  | -11,62 | 0,00 | -10,55 | 0,00 | -11,27 | 0,00 | probable alkyl hydroperoxide reductase | Adaptation Protection |
| PA0849_trxB2 |  |  | -2,81 | 0,00 | -3,04 | 0,21 | -2,89 | 0,00 | thioredoxin reductase 2 | Nucleotide biosynthesis and metabolism |
| PA0852_cpbD |  | ***lasR+rhlR*** | -6,14 | 0,00 | -5,20 | 0,00 | -3,97 | 0,00 | chitin-binding protein CbpD precursor | Secreted Factors (toxins enzymes alginate) |
| PA0996_pqsA | ***mvfR*** | ***lasR+rhlR*** | -5,33 | 0,00 | -3,90 | 0,21 | -9,55 | 0,00 | probable coenzyme A ligase | Biosynthesis of cofactors prosthetic groups and carriers |
| PA0997_pqsB | ***mvfR*** | ***lasR+rhlR*** | -6,98 | 0,00 | -4,63 | 0,00 | -17,55 | 0,00 | Homologous to beta-keto-acyl-acyl-carrier protein synthase | Biosynthesis of cofactors prosthetic groups and carriers |
| PA0998_pqsC | ***mvfR*** | ***lasR+rhlR*** | -6,74 | 0,00 | -4,92 | 0,00 | -22,60 | 0,00 | Homologous to beta-keto-acyl-acyl-carrier protein synthase | Biosynthesis of cofactors prosthetic groups and carriers |
| PA0999_pqsD | ***mvfR*** | ***lasR+rhlR*** | -6,81 | 0,00 | -4,86 | 0,00 | -16,98 | 0,00 | 3-oxoacyl-[acyl-carrier-protein] synthase III | Biosynthesis of cofactors prosthetic groups and carriers |
| PA1000_pqsE | ***mvfR*** | ***lasR+rhlR*** | -6,66 | 0,00 | -4,62 | 0,00 | -15,71 | 0,00 | Quinolone signal response protein | Biosynthesis of cofactors prosthetic groups and carriers |
| PA1001_phnA | ***mvfR*** | ***lasR+rhlR*** | -6,52 | 0,00 | -4,81 | 0,00 | -15,81 | 0,00 | anthranilate synthase component I | Adaptation Protection |
| PA1002_phnB | ***mvfR*** | ***lasR+rhlR*** | -6,79 | 0,00 | -5,91 | 0,00 | -13,55 | 0,00 | anthranilate synthase component II | Amino acid biosynthesis and metabolism |
| PA1202 |  |  | -3,58 | 0,00 | -3,72 | 0,00 | -3,11 | 0,00 | probable hydrolase | Putative enzymes |
| PA1211 |  |  | -2,25 | 0,94 | -2,63 | 0,13 | -2,65 | 0,00 | hypothetical protein | Hypothetical, unclassified, unknown |
| PA1212 |  | ***lasR+rhlR*** | -2,48 | 0,00 | -2,99 | 0,00 | -3,12 | 0,00 | probable major facilitator superfamily (MFS) transporter | Membrane proteins |
| PA1213 |  |  | -2,68 | 0,76 | -4,36 | 0,00 | -5,57 | 0,00 | hypothetical protein | Hypothetical, unclassified, unknown |
| PA1214 |  | ***lasR+rhlR*** | -4,27 | 0,00 | -6,00 | 0,00 | -9,55 | 0,00 | hypothetical protein | Hypothetical, unclassified, unknown |
| PA1215 |  | ***lasR+rhlR*** | -4,17 | 0,00 | -6,09 | 0,00 | -10,19 | 0,00 | hypothetical protein | Hypothetical, unclassified, unknown |
| PA1216 | ***mvfR*** | ***lasR+rhlR*** | -6,20 | 0,00 | -7,81 | 0,00 | -12,51 | 0,00 | hypothetical protein | Hypothetical, unclassified, unknown |
| PA1217 |  | ***lasR+rhlR*** | -5,79 | 0,00 | -8,44 | 0,00 | -14,73 | 0,00 | probable 2-isopropylmalate synthase | Amino acid biosynthesis and metabolism |
| PA1218 |  | ***lasR+rhlR*** | -6,46 | 0,00 | -6,59 | 0,00 | -9,46 | 0,00 | hypothetical protein | Hypothetical, unclassified, unknown |
| PA1219 |  | ***lasR+rhlR*** | -2,10 | 0,00 | -3,19 | 0,00 | -3,61 | 0,00 | hypothetical protein | Hypothetical, unclassified, unknown |
| PA1220 |  |  | -2,49 | 0,00 | -3,19 | 0,00 | -3,43 | 0,00 | hypothetical protein | Hypothetical, unclassified, unknown |
| PA1221 |  | ***lasR+rhlR*** | -2,10 | 4,08 | -3,09 | 0,21 | -3,33 | 0,00 | hypothetical protein | Hypothetical, unclassified, unknown |
| PA1240 |  |  | -2,10 | 5,81 | -2,68 | 0,21 | -3,05 | 0,00 | probable enoyl-CoA hydratase/isomerase | Putative enzymes |
| PA1324 |  | ***lasR+rhlR*** | -2,14 | 4,08 | -2,37 | 0,22 | -2,25 | 0,00 | hypothetical protein | Hypothetical, unclassified, unknown |
| PA1344 |  |  | -2,73 | 0,00 | -2,52 | 0,00 | -3,19 | 0,00 | probable short-chain dehydrogenase | Putative enzymes |
| PA1349 |  |  | -2,61 | 0,00 | -3,02 | 0,21 | -2,22 | 0,00 | conserved hypothetical protein | Hypothetical, unclassified, unknown |
| PA1350 |  |  | -2,84 | 0,00 | -3,37 | 0,00 | -2,84 | 0,00 | hypothetical protein | Hypothetical, unclassified, unknown |
| PA1356 |  |  | -2,14 | 2,77 | -2,85 | 0,21 | -2,03 | 0,03 | hypothetical protein | Hypothetical, unclassified, unknown |
| PA1784 |  | ***lasR+rhlR*** | -5,23 | 0,00 | -6,03 | 0,21 | -3,30 | 0,00 | hypothetical protein | Hypothetical, unclassified, unknown |
| PA1871_lasA |  | ***lasR+rhlR*** | -6,15 | 0,00 | -10,83 | 0,00 | -3,74 | 0,00 | LasA protease precursor | Secreted Factors (toxins enzymes alginate) |
| PA1874 |  | ***lasR+rhlR*** | -10,83 | 0,00 | -25,99 | 0,00 | -28,69 | 0,00 | hypothetical protein | Hypothetical, unclassified, unknown |
| PA1875 |  | ***lasR+rhlR*** | -9,16 | 0,00 | -15,07 | 0,00 | -17,60 | 0,00 | probable outer membrane protein precursor | Protein secretion/export apparatus |
| PA1876 |  |  | -14,05 | 0,00 | -22,81 | 0,00 | -22,08 | 0,00 | probable ATP-binding/permease fusion ABC transporter | Secreted Factors (toxins enzymes alginate) |
| PA1877 |  | ***lasR+rhlR*** | -5,50 | 0,00 | -9,15 | 0,00 | -9,46 | 0,00 | probable secretion protein | Protein secretion/export apparatus |
| PA1880 |  |  | -2,36 | 0,76 | -3,46 | 0,00 | -2,35 | 0,00 | probable oxidoreductase | Putative enzymes |
| PA1881 |  | ***lasR+rhlR*** | -3,16 | 0,00 | -3,47 | 0,00 | -2,42 | 0,00 | probable oxidoreductase | Putative enzymes |
| PA1888 |  | ***lasR+rhlR*** | -3,85 | 0,00 | -6,49 | 0,00 | -2,63 | 0,00 | hypothetical protein | Hypothetical, unclassified, unknown |
| PA1914 | ***mvfR*** | ***lasR+rhlR*** | -23,43 | 0,00 | -44,72 | 0,00 | -44,02 | 0,00 | conserved hypothetical protein | Putative enzymes |
| PA1927_metE |  | ***lasR+rhlR*** | -3,35 | 2,74 | -4,34 | 0,22 | -4,31 | 0,00 | 5-methyltetrahydropteroyltriglutamate-homocysteineS-methyltransferase | Amino acid biosynthesis and metabolism |
| PA1930 |  | ***lasR+rhlR*** | -2,49 | 0,00 | -4,21 | 0,21 | -2,15 | 0,00 | probable chemotaxis transducer | Adaptation Protection Chemotaxis |
| PA1985_pqqA |  |  | -2,43 | 4,08 | -2,29 | 0,35 | -3,80 | 0,00 | pyrroloquinoline quinone biosynthesis protein A | Biosynthesis of cofactors prosthetic groups and carriers |
| PA2003_bdhA |  |  | -3,64 | 0,00 | -4,25 | 0,00 | -2,67 | 0,00 | 3-hydroxybutyrate dehydrogenase | Carbon compound catabolism |
| PA2004 |  |  | -5,04 | 0,00 | -4,12 | 0,00 | -4,07 | 0,00 | conserved hypothetical protein | Membrane proteins |
| PA2030 |  | ***lasR+rhlR*** | -5,59 | 0,00 | -5,99 | 0,00 | -6,08 | 0,00 | hypothetical protein | Hypothetical, unclassified, unknown |
| PA2031_i | ***mvfR*** | ***lasR+rhlR*** | -5,62 | 0,00 | -6,25 | 0,00 | -4,77 | 0,00 | hypothetical protein | Hypothetical, unclassified, unknown |
| PA2067 | ***mvfR*** | ***lasR+rhlR*** | -3,11 | 0,76 | -2,71 | 0,00 | -3,16 | 0,00 | probable hydrolase | Putative enzymes |
| PA2069 | ***mvfR*** | ***lasR+rhlR*** | -4,01 | 0,00 | -2,77 | 0,21 | -4,05 | 0,00 | probable carbamoyl transferase | Putative enzymes |
| PA2134 | ***mvfR*** | ***lasR+rhlR*** | -2,30 | 0,00 | -2,88 | 0,00 | -2,29 | 0,00 | hypothetical protein | Hypothetical, unclassified, unknown |
| PA2147_katE |  | ***lasR+rhlR*** | -2,08 | 4,08 | -3,38 | 0,21 | -3,66 | 0,00 | catalase HPII | Adaptation Protection |
| PA2151 |  | ***lasR+rhlR*** | -2,17 | 4,08 | -3,71 | 0,21 | -3,59 | 0,00 | conserved hypothetical protein | Hypothetical, unclassified, unknown |
| PA2152 |  | ***lasR+rhlR*** | -2,89 | 4,08 | -3,69 | 0,21 | -3,80 | 0,00 | probable trehalose synthase | Putative enzymes |
| PA2274 | ***mvfR*** | ***lasR+rhlR*** | -11,40 | 0,00 | -5,83 | 0,00 | -18,23 | 0,00 | hypothetical protein | Hypothetical, unclassified, unknown |
| PA2290_gcd |  |  | -2,01 | 2,77 | -3,14 | 0,00 | -2,19 | 0,00 | glucose dehydrogenase | Carbon compound catabolism |
| PA2299 | ***mvfR*** |  | -2,13 | 0,76 | -2,14 | 0,21 | -2,30 | 0,00 | conserved hypothetical protein | Membrane proteins |
| PA2300_chiC | ***mvfR*** | ***lasR+rhlR*** | -11,95 | 0,00 | -13,11 | 0,00 | -8,69 | 0,00 | chitinase | Carbon compound catabolism |
| PA2330 |  | ***lasR+rhlR*** | -2,89 | 0,76 | -2,20 | 0,28 | -3,96 | 0,05 | hypothetical protein | Hypothetical, unclassified, unknown |
| PA2331 | ***mvfR*** | ***lasR+rhlR*** | -2,93 | 0,00 | -2,10 | 0,75 | -3,37 | 0,07 | hypothetical protein | Membrane proteins |
| PA2365 |  | ***lasR+rhlR*** | -2,87 | 0,00 | -3,80 | 0,22 | -4,00 | 0,00 | conserved hypothetical protein | Hypothetical, unclassified, unknown |
| PA2366 |  | ***lasR+rhlR*** | -2,64 | 0,94 | -3,92 | 0,21 | -2,62 | 0,00 | conserved hypothetical protein | Hypothetical, unclassified, unknown |
| PA2368_i |  | ***lasR+rhlR*** | -2,03 | 2,74 | -3,35 | 0,00 | -2,00 | 0,00 | hypothetical protein | Hypothetical, unclassified, unknown |
| PA2381 |  |  | -2,72 | 0,00 | -2,03 | 0,21 | -3,89 | 0,00 | hypothetical protein | Hypothetical, unclassified, unknown |
| PA2414 |  | ***lasR+rhlR*** | -2,24 | 2,77 | -4,17 | 0,00 | -2,59 | 0,00 | L-sorbosone dehydrogenase | Carbon compound catabolism |
| PA2448 |  | ***lasR+rhlR*** | -4,18 | 0,00 | -5,22 | 0,00 | -5,23 | 0,00 | hypothetical protein | Hypothetical, unclassified, unknown |
| PA2504 |  |  | -3,02 | 0,00 | -2,86 | 0,13 | -2,61 | 0,00 | hypothetical protein | Hypothetical, unclassified, unknown |
| PA2564 |  | ***lasR+rhlR*** | -2,29 | 0,00 | -3,02 | 0,00 | -2,04 | 0,00 | hypothetical protein | Hypothetical, unclassified, unknown |
| PA2565 |  | ***lasR+rhlR*** | -2,07 | 2,77 | -2,94 | 0,07 | -2,15 | 0,00 | hypothetical protein | Hypothetical, unclassified, unknown |
| PA2566 |  | ***lasR+rhlR*** | -3,38 | 0,00 | -6,02 | 0,00 | -3,07 | 0,00 | conserved hypothetical protein | Hypothetical, unclassified, unknown |
| PA2570_lecA | ***mvfR*** | ***lasR+rhlR*** | -4,87 | 0,00 | -5,14 | 0,00 | -4,98 | 0,00 | PA-I galactophilic lectin | Adaptation Protection |
| PA2571 |  |  | -2,34 | 0,00 | -3,02 | 0,00 | -2,41 | 0,00 | probable two-component sensor | Two-component regulatory systems |
| PA2699 |  |  | -2,75 | 3,44 | -3,59 | 0,21 | -2,64 | 0,03 | hypothetical protein | Hypothetical, unclassified, unknown |
| PA2717_cpo |  | ***lasR+rhlR*** | -3,92 | 0,00 | -4,47 | 0,00 | -3,27 | 0,00 | chloroperoxidase precursor | Central intermediary metabolism |
| PA2815 |  |  | -2,24 | 2,77 | -2,57 | 0,00 | -2,67 | 0,00 | probable acyl-CoA dehydrogenase | Putative enzymes |
| PA2826 |  |  | -2,73 | 1,87 | -2,26 | 0,75 | -2,30 | 0,05 | probable glutathione peroxidase | Adaptation Protection |
| PA2868_i |  |  | -3,35 | 0,00 | -3,35 | 0,00 | -2,34 | 0,00 | hypothetical protein | Membrane proteins |
| PA2915 |  |  | -2,35 | 1,87 | -2,25 | 0,00 | -2,13 | 0,00 | hypothetical protein | Hypothetical, unclassified, unknown |
| PA2927 |  | ***lasR+rhlR*** | -3,77 | 1,87 | -3,04 | 0,13 | -2,73 | 0,00 | hypothetical protein | Hypothetical, unclassified, unknown |
| PA2939 |  | ***lasR+rhlR*** | -4,70 | 0,94 | -7,04 | 0,00 | -6,34 | 0,00 | probable aminopeptidase | Secreted Factors (toxins enzymes alginate) |
| PA3032 |  | ***lasR+rhlR*** | -6,95 | 0,00 | -15,61 | 0,00 | -5,93 | 0,00 | cytochrome c Snr1 | Energy metabolism |
| PA3123 |  |  | -2,72 | 0,00 | -2,68 | 0,00 | -2,34 | 0,00 | conserved hypothetical protein | Hypothetical, unclassified, unknown |
| PA3237 |  |  | -6,67 | 0,00 | -7,61 | 0,13 | -6,78 | 0,00 | hypothetical protein | Hypothetical, unclassified, unknown |
| PA3250 |  |  | -3,43 | 0,94 | -3,38 | 0,21 | -4,17 | 0,00 | hypothetical protein | Hypothetical, unclassified, unknown |
| PA3287 |  |  | -10,43 | 0,00 | -13,11 | 0,00 | -10,04 | 0,00 | conserved hypothetical protein | Hypothetical, unclassified, unknown |
| PA3340 |  |  | -2,17 | 0,94 | -2,50 | 0,00 | -2,45 | 0,00 | hypothetical protein | Membrane proteins |
| PA3415 |  |  | -2,68 | 0,00 | -4,01 | 0,00 | -2,03 | 0,00 | probable dihydrolipoamide acetyltransferase | Energy metabolism |
| PA3416 |  | ***lasR+rhlR*** | -3,17 | 0,00 | -5,04 | 0,00 | -2,72 | 0,00 | probable pyruvate dehydrogenase E1 component, beta chain | Energy metabolism |
| PA3417 |  |  | -2,33 | 0,00 | -4,48 | 0,00 | -2,61 | 0,00 | probable pyruvate dehydrogenase E1 component, alpha subunit | Energy metabolism |
| PA3460 |  |  | -2,45 | 1,20 | -2,68 | 0,22 | -2,93 | 0,00 | probable acetyltransferase | Putative enzymes |
| PA3677 |  | ***lasR+rhlR*** | -2,56 | 0,00 | -2,85 | 0,21 | -2,59 | 0,00 | probable Resistance-Nodulation-Cell Division (RND) efflux membrane fusion protein precursor | Transport of small molecules |
| PA3688 |  | ***lasR+rhlR*** | -3,38 | 0,00 | -3,66 | 0,00 | -2,07 | 0,00 | hypothetical protein | Hypothetical, unclassified, unknown |
| PA3718 |  |  | -4,18 | 0,00 | -3,85 | 0,00 | -3,81 | 0,00 | probable major facilitator superfamily (MFS) transporter | Membrane proteins |
| PA3723 |  |  | -2,38 | 0,76 | -3,57 | 0,00 | -2,34 | 0,00 | probable FMN oxidoreductase | Putative enzymes |
| PA3724_lasB |  | ***lasR+rhlR*** | -2,71 | 3,89 | -3,10 | 0,21 | -2,44 | 0,00 | elastase LasB | Secreted Factors (toxins enzymes alginate) |
| PA3734 |  | ***lasR+rhlR*** | -2,70 | 0,00 | -2,78 | 0,13 | -2,99 | 0,00 | hypothetical protein | Hypothetical, unclassified, unknown |
| PA3924 |  |  | -2,71 | 0,00 | -2,74 | 0,00 | -2,31 | 0,00 | probable medium-chain acyl-CoA ligase | Fatty acid and phospholipid metabolism |
| PA3929_cioB |  |  | -2,18 | 1,87 | -2,44 | 0,22 | -5,46 | 0,00 | cyanide insensitive terminal oxidase | Energy metabolism |
| PA3930_cioA |  |  | -3,90 | 0,94 | -2,62 | 0,28 | -9,70 | 0,00 | cyanide insensitive terminal oxidase | Energy metabolism |
| PA4015 |  |  | -2,97 | 2,74 | -2,25 | 0,21 | -2,06 | 0,00 | conserved hypothetical protein | Hypothetical, unclassified, unknown |
| PA4078 | ***mvfR*** | ***lasR+rhlR*** | -6,28 | 0,00 | -8,61 | 0,00 | -6,41 | 0,00 | probable nonribosomal peptide synthetase | Adaptation Protection |
| PA4079 | ***mvfR*** |  | -2,84 | 0,00 | -2,44 | 0,00 | -2,30 | 0,00 | probable dehydrogenase | Putative enzymes |
| PA4141 | ***mvfR*** | ***lasR+rhlR*** | -4,76 | 0,00 | -4,52 | 0,00 | -2,20 | 0,03 | hypothetical protein | Hypothetical, unclassified, unknown |
| PA4175 |  | ***lasR+rhlR*** | -4,53 | 0,00 | -4,27 | 0,35 | -5,31 | 0,00 | Pvds-regulated endoprotease, lysyl class | Putative enzymes |
| PA4205_mexG | ***mvfR*** | ***lasR+rhlR*** | -23,88 | 0,00 | -10,88 | 0,00 | -95,80 | 0,00 | hypothetical protein | Membrane proteins |
| PA4206_mexH | ***mvfR*** | ***lasR+rhlR*** | -15,51 | 0,00 | -9,34 | 0,00 | -46,72 | 0,00 | probable Resistance-Nodulation-Cell Division (RND) efflux membrane fusion protein precursor | Transport of small molecules |
| PA4207_mexI | ***mvfR*** | ***lasR+rhlR*** | -9,58 | 0,00 | -7,99 | 0,00 | -45,44 | 0,00 | probable Resistance-Nodulation-Cell Division (RND) efflux transporter | Membrane proteins |
| PA4208_opmD | ***mvfR*** | ***lasR+rhlR*** | -10,08 | 0,00 | -6,91 | 0,00 | -27,83 | 0,00 | probable outer membrane protein precursor | Membrane proteins |
| PA4210_phzA1 | ***mvfR*** | ***lasR+rhlR*** | -5,65 | 0,00 | -4,66 | 0,00 | -10,65 | 0,00 | probable phenazine biosynthesis protein | Secreted Factors (toxins enzymes alginate) |
| PA4211_phzB1 | ***mvfR*** | ***lasR+rhlR*** | -4,13 | 0,94 | -3,31 | 0,00 | -7,57 | 0,00 | probable phenazine biosynthesis protein | Secreted Factors (toxins enzymes alginate) |
| PA4212_phzC1 | ***mvfR*** | ***lasR+rhlR*** | -3,02 | 0,00 | -2,85 | 0,00 | -8,83 | 0,00 | phenazine biosynthesis protein PhzC | Secreted Factors (toxins enzymes alginate) |
| PA4213_phzD1 | ***mvfR*** | ***lasR+rhlR*** | -4,50 | 0,94 | -3,47 | 0,00 | -10,78 | 0,00 | phenazine biosynthesis protein PhzD | Secreted Factors (toxins enzymes alginate) |
| PA4214_phzE1 | ***mvfR*** | ***lasR+rhlR*** | -3,45 | 0,00 | -2,97 | 0,13 | -9,47 | 0,00 | phenazine biosynthesis protein PhzE | Secreted Factors (toxins enzymes alginate) |
| PA4215_phzF1 | ***mvfR*** | ***lasR+rhlR*** | -3,60 | 0,00 | -3,17 | 0,00 | -10,42 | 0,00 | probable phenazine biosynthesis protein | Secreted Factors (toxins enzymes alginate) |
| PA4216_phzG1 | ***mvfR*** | ***lasR+rhlR*** | -3,26 | 0,00 | -2,56 | 0,13 | -7,08 | 0,00 | probable pyridoxamine 5'-phosphate oxidase | Secreted Factors (toxins enzymes alginate) |
| PA4227_pchR |  |  | -2,40 | 0,76 | -2,74 | 0,00 | -2,97 | 0,00 | transcriptional regulator PchR | Transcriptional regulators |
| PA4293 |  | ***lasR+rhlR*** | -4,11 | 0,00 | -6,71 | 0,00 | -7,25 | 0,00 | probable two-component sensor | Two-component regulatory systems |
| PA4294 |  | ***lasR+rhlR*** | -2,24 | 1,87 | -2,86 | 0,13 | -2,83 | 0,00 | hypothetical protein | Hypothetical, unclassified, unknown |
| PA4296 |  | ***lasR+rhlR*** | -3,57 | 2,77 | -2,90 | 0,00 | -5,05 | 0,00 | probable two-component response regulator | Transcriptional regulators |
| PA4298 |  | ***lasR+rhlR*** | -5,33 | 0,00 | -7,38 | 0,00 | -6,88 | 0,00 | hypothetical protein | Hypothetical, unclassified, unknown |
| PA4299 |  | ***lasR+rhlR*** | -4,48 | 0,00 | -6,62 | 0,00 | -7,38 | 0,00 | hypothetical protein | Hypothetical, unclassified, unknown |
| PA4300 |  | ***lasR+rhlR*** | -2,90 | 0,76 | -4,20 | 0,00 | -5,04 | 0,00 | hypothetical protein | Membrane proteins |
| PA4302 |  | ***lasR+rhlR*** | -4,33 | 0,00 | -5,29 | 0,00 | -6,84 | 0,00 | probable type II secretion system protein | Protein secretion/export apparatus |
| PA4303 |  | ***lasR+rhlR*** | -2,87 | 0,00 | -3,59 | 0,00 | -4,20 | 0,00 | hypothetical protein | Hypothetical, unclassified, unknown |
| PA4304 |  | ***lasR+rhlR*** | -4,02 | 0,00 | -6,19 | 0,00 | -8,29 | 0,00 | probable type II secretion system protein | Protein secretion/export apparatus |
| PA4305 |  | ***lasR+rhlR*** | -3,73 | 0,00 | -5,10 | 0,00 | -4,82 | 0,00 | hypothetical protein | Hypothetical, unclassified, unknown |
| PA4306 |  | ***lasR+rhlR*** | -7,39 | 0,00 | -13,79 | 0,00 | -23,82 | 0,00 | hypothetical protein | Hypothetical, unclassified, unknown |
| PA4311 |  | ***lasR+rhlR*** | -2,39 | 0,94 | -2,76 | 0,00 | -2,66 | 0,00 | conserved hypothetical protein | Hypothetical, unclassified, unknown |
| PA4312 |  |  | -2,23 | 3,44 | -2,56 | 0,00 | -2,49 | 0,00 | conserved hypothetical protein | Hypothetical, unclassified, unknown |
| PA4362 |  |  | -2,32 | 0,00 | -3,06 | 0,00 | -2,49 | 0,00 | hypothetical protein | Hypothetical, unclassified, unknown |
| PA4384 |  | ***lasR+rhlR*** | -2,42 | 4,08 | -3,43 | 0,00 | -3,91 | 0,00 | hypothetical protein | Hypothetical, unclassified, unknown |
| PA4607 |  |  | -3,65 | 0,76 | -2,75 | 0,00 | -2,14 | 0,00 | hypothetical protein | Hypothetical, unclassified, unknown |
| PA4613_katB |  |  | -4,54 | 0,00 | -6,48 | 0,21 | -7,02 | 0,00 | catalase | Adaptation Protection |
| PA4614_mscL |  |  | -2,64 | 2,74 | -2,05 | 0,35 | -2,29 | 0,00 | conductance mechanosensitive channel | Membrane proteins |
| PA4641 |  |  | -2,17 | 2,77 | -3,16 | 0,21 | -2,21 | 0,00 | still frameshift hypothetical protein | Hypothetical, unclassified, unknown |
| PA4648 |  | ***lasR+rhlR*** | -5,12 | 0,00 | -7,49 | 0,00 | -6,13 | 0,00 | hypothetical protein | Hypothetical, unclassified, unknown |
| PA4651 |  | ***lasR+rhlR*** | -4,71 | 0,00 | -8,33 | 0,00 | -8,26 | 0,00 | probable pili assembly chaperone | Motility & Attachment |
| PA4653 |  |  | -3,28 | 0,00 | -4,04 | 0,21 | -3,83 | 0,00 | hypothetical protein | Hypothetical, unclassified, unknown |
| PA4733_acsB |  |  | -2,53 | 5,81 | -2,10 | 0,21 | -2,08 | 0,00 | acetyl-coenzyme A synthetase | Carbon compound catabolism |
| PA4876_osmE | ***mvfR*** | ***lasR+rhlR*** | -2,50 | 0,00 | -2,27 | 0,28 | -2,05 | 0,00 | osmotically inducible lipoprotein OsmE | Membrane proteins |
| PA4925 |  | ***lasR+rhlR*** | -3,10 | 0,00 | -5,41 | 0,00 | -3,36 | 0,00 | conserved hypothetical protein | Hypothetical, unclassified, unknown |
| PA5058_phaC2 |  | ***lasR+rhlR*** | -3,97 | 0,00 | -5,45 | 0,21 | -4,50 | 0,00 | poly(3-hydroxyalkanoic acid) synthase 2 | Central intermediary metabolism |
| PA5059 |  | ***lasR+rhlR*** | -3,61 | 0,00 | -3,99 | 0,13 | -3,03 | 0,00 | probable transcriptional regulator | Transcriptional regulators |
| PA5101 |  |  | -2,09 | 0,94 | -2,52 | 0,00 | -2,11 | 0,00 | hypothetical protein | Hypothetical, unclassified, unknown |
| PA5213_gcvP1 |  |  | -2,70 | 0,00 | -3,83 | 0,00 | -2,43 | 0,00 | glycine cleavage system protein P1 | Central intermediary metabolism |
| PA5220 |  | ***lasR+rhlR*** | -2,67 | 3,44 | -2,55 | 0,00 | -2,59 | 0,00 | hypothetical protein | Hypothetical, unclassified, unknown |
| PA5313 |  |  | -2,19 | 1,20 | -2,01 | 0,00 | -3,88 | 0,00 | probable pyridoxal-dependent aminotransferase | Putative enzymes |
| PA5482 | ***mvfR*** | ***lasR+rhlR*** | -2,27 | 3,44 | -2,04 | 0,84 | -3,29 | 0,00 | hypothetical protein | Membrane proteins |
| PA5496 |  |  | -2,61 | 1,20 | -2,33 | 0,21 | -2,11 | 0,00 | hypothetical protein | Hypothetical, unclassified, unknown |
| PA5497 |  |  | -2,33 | 5,81 | -2,09 | 0,00 | -2,80 | 0,00 | hypothetical protein | Hypothetical, unclassified, unknown |
| **Genes activated by AA analogs** | | |  |  |  |  |  |  |  |  |
|  |  |  |  |  |  |  |  |  |  |  |
| PA0509_nirN |  | ***lasR+rhlR*** | 2,85 | 0,00 | 2,61 | 0,12 | 2,69 | 0,00 | probable c-type cytochrome | Biosynthesis of cofactors prosthetic groups and carriers |
| PA0510 |  | ***lasR+rhlR*** | 3,99 | 0,00 | 2,52 | 0,12 | 2,88 | 0,00 | probable uroporphyrin-III c-methyltransferase | Biosynthesis of cofactors prosthetic groups and carriers |
| PA0511_nirJ |  |  | 2,98 | 4,66 | 3,01 | 0,12 | 4,00 | 0,00 | heme d1 biosynthesis protein NirJ | Biosynthesis of cofactors prosthetic groups and carriers |
| PA0512 |  | ***lasR+rhlR*** | 3,82 | 1,87 | 3,96 | 0,12 | 4,97 | 0,00 | conserved hypothetical protein | Biosynthesis of cofactors prosthetic groups and carriers |
| PA0514_nirL |  |  | 3,10 | 0,00 | 2,43 | 0,20 | 3,11 | 0,00 | heme d1 biosynthesis protein NirL | Hypothetical, unclassified, unknown |
| PA0515 |  |  | 3,79 | 0,00 | 3,02 | 0,12 | 4,55 | 0,00 | probable transcriptional regulator | Biosynthesis of cofactors prosthetic groups and carriers |
| PA0516_nirF |  |  | 3,44 | 3,44 | 2,53 | 0,20 | 3,43 | 0,00 | heme d1 biosynthesis protein NirF | Energy metabolism |
| PA0518_nirM |  |  | 5,32 | 0,00 | 3,78 | 0,12 | 6,26 | 0,00 | cytochrome c-551 precursor | Biosynthesis of cofactors prosthetic groups and carriers |
| PA0526 |  |  | 7,22 | 0,00 | 4,01 | 0,12 | 12,27 | 0,00 | hypothetical protein | Hypothetical, unclassified, unknown |
| PA2511 |  |  | 5,13 | 0,00 | 4,41 | 0,00 | 3,28 | 0,00 | probable transcriptional regulator | Transcriptional regulators |
| PA2512_antA |  |  | 249,03 | 0,00 | 267,86 | 0,00 | 152,15 | 0,00 | anthranilate dioxygenase large subunit | Carbon compound catabolism |
| PA2513_antB |  |  | 407,42 | 0,00 | 433,58 | 0,00 | 269,29 | 0,00 | anthranilate dioxygenase small subunit | Carbon compound catabolism |
| PA2514_antC |  |  | 254,06 | 0,00 | 241,21 | 0,00 | 123,10 | 0,00 | anthranilate dioxygenase reductase | Carbon compound catabolism |
| PA2630 |  |  | 3,63 | 5,81 | 3,90 | 0,00 | 4,44 | 0,00 | conserved hypothetical protein | Hypothetical, unclassified, unknown |
| PA2682 |  |  | 23,11 | 0,00 | 26,99 | 0,00 | 8,29 | 0,00 | conserved hypothetical protein | Putative enzymes |
| PA3327 |  | ***lasR+rhlR*** | 6,30 | 0,00 | 3,98 | 0,00 | 3,24 | 0,00 | probable non-ribosomal peptide synthetase | Adaptation Protection |
| PA3328 |  | ***lasR+rhlR*** | 4,15 | 1,87 | 3,20 | 0,12 | 3,03 | 0,00 | probable FAD-dependent monooxygenase | Putative enzymes |
| PA3329 |  | ***lasR+rhlR*** | 3,88 | 0,00 | 2,69 | 0,12 | 2,52 | 0,00 | hypothetical protein | Hypothetical, unclassified, unknown |
| PA3332 |  | ***lasR+rhlR*** | 2,70 | 1,20 | 2,20 | 0,12 | 2,09 | 0,00 | conserved hypothetical protein | Hypothetical, unclassified, unknown |
| PA3458 |  |  | 4,02 | 0,00 | 2,87 | 0,12 | 3,72 | 0,00 | probable transcriptional regulator | Transcriptional regulators |
| PA3743_trmD |  |  | 2,71 | 1,87 | 2,61 | 0,12 | 2,36 | 0,00 | tRNA (guanine-N1)-methyltransferase | Transcription RNA processing and degradation |
| PA3911 |  | ***lasR+rhlR*** | 2,31 | 3,89 | 2,22 | 0,20 | 2,44 | 0,00 | conserved hypothetical protein | Hypothetical, unclassified, unknown |
| PA3973 | ***mvfR*** |  | 3,61 | 2,74 | 2,61 | 0,28 | 4,54 | 0,00 | probable transcriptional regulator | Transcriptional regulators |
| PA4321 |  |  | 2,30 | 2,77 | 2,07 | 0,00 | 2,03 | 0,00 | hypothetical protein | Hypothetical, unclassified, unknown |
| PA4480_mreC |  |  | 2,14 | 3,89 | 2,16 | 0,12 | 2,41 | 0,00 | rod shape-determining protein MreC | Cell wall / LPS / capsule Cell division |
| PA4494 |  |  | 2,40 | 0,00 | 2,65 | 0,00 | 3,14 | 0,00 | probable two-component sensor | Two-component regulatory systems |
| PA4542_clpB | ***mvfR*** |  | 3,42 | 4,66 | 2,24 | 0,28 | 2,12 | 0,00 | ClpB protein | Translation, post-translational modification, degradation |
| PA4596_ilvI |  |  | 3,11 | 5,15 | 2,63 | 1,09 | 5,21 | 0,00 | acetolactate synthase large subunit | Biosynthesis of cofactors, prosthetic groups and carriers |
| PA4685 |  |  | 2,16 | 1,20 | 2,60 | 0,12 | 2,33 | 0,00 | hypothetical protein | Hypothetical, unclassified, unknown |
| PA4853_fis |  |  | 2,39 | 3,44 | 3,53 | 0,12 | 3,70 | 0,00 | DNA-binding protein Fis | Transcriptional regulators |
| PA4934_rpsR |  |  | 2,41 | 5,15 | 3,16 | 0,07 | 2,09 | 0,00 | 30S ribosomal protein S18 | Translation, post-translational modification, degradation |
| PA5367_pstA |  |  | 2,96 | 2,77 | 2,21 | 0,12 | 2,94 | 0,00 | membrane protein component of ABC phosphate transporter | Membrane proteins |

(i) The ratios correspond to the fold change in differential gene expression for PA14 cells grown in 6 mM 6FABA, 6 mM 6CABA or 1.5 mM 4CABA versus the control cells, at OD=2.5. Q-values, outputed from SAM, are the estimated false discovery rates. (-), ratio is lower in response to AA analog growth versus the control.

(ii) PA numbers and annotation are from http:/www.pseudomonas.com

(iii) Genes identified previously as MvfR-dependent [25] are highlighted in gray. *lasR/rhlR*-dependent genes have been previously described [19,41,42]

(iv) The Affymetrix *P. aeruginosa* microarray contains probe sets for only one copy of each *phzA-G* gene, labeled PA4210 (*phzA1*), PA4211 (*phzB1*) and PA1901 through PA1905 (*phzC2D2E2F2G2*). PA1901-PA1905 were previously reassigned to their corresponding phz1 genes [25].
