# Supplementary material for: Inhibitors of Pathogen Intercellular Signals as Selective Anti-Infective Compounds
Source: PLoS Pathog. 2007 Sep 14;3(9):e126. doi: 10.1371/journal.ppat.0030126 (PMC2323289; doi:10.1371/journal.ppat.0030126)
Supplement: Table S2 — (297 KB DOC) [file ppat.0030126.st002.doc]

**Table S2: Differential expression levels of MvfR positively and negatively regulated genes in response to 6FABA, 6CABA, or 4CABA**

|  |  | **6FABA** | | | **6CABA** | | **4CABA** | | **Function** |
| --- | --- | --- | --- | --- | --- | --- | --- | --- | --- |
|  | **Positively regulated by MvfR (122 genes) ^(a)^** | | | | | | | |  |
|  | PA0038 |  | | -1.85 | | |  | | Hypothetical protein |
|  | PA0051_phzH | -10.26 | | -7.18 | | | -1.69 | | potential phenazine-modifying enzyme |
|  | PA0122 | -3.4 | | -3.42 | | | -2.74 | | Hypothetical protein |
|  | PA0200 |  | |  | | | 2.19 | | Hypothetical protein |
|  | PA0283_sbp |  | |  | | | 1.97 | | Sulfate binding protein |
|  | PA0284 |  | |  | | | 2.87 | | Hypothetical protein |
|  | PA0336_ygdP |  | |  | | |  | | Nudix hydrolase |
|  | PA0355_pfpI | -2.20 | | -2.08 | | | -2.35 | | protease PfpI |
|  | PA0387 |  | |  | | |  | | Hypothetical protein |
|  | PA0456 |  | | 1.75 | | | 1.84 | | Probable cold shock protein |
|  | PA0567 |  | |  | | | -2.08 | | Hypothetical protein |
|  | PA0589 |  | |  | | | 1.76 | | Hypothetical protein |
|  | PA0590_apaH |  | |  | | |  | | Bis(5’-nucleosyl)-tetraphosphatase |
|  | PA0591 |  | |  | | |  | | Hypothetical protein |
|  | PA0762_algU |  | | 1.53 | | | 2.59 | | EDF sigma factor |
|  | PA0807 |  | |  | | | -2.44 | | Hypothetical protein |
|  | PA0905_rsmA |  | |  | | | 3.27 | | Global post-transcriptional regulator |
|  | PA0996_pqsA | -5.33 | | -3.90 | | | -9.55 | | probable coenzyme A ligase |
|  | PA0997_pqsB | -6.98 | | -4.63 | | | -17.55 | | Homologous to beta-keto-acyl-acyl-carrier protein synthase |
|  | PA0998_pqsC | -6.74 | | -4.92 | | | -22.6 | | Homologous to beta-keto-acyl-acyl-carrier protein synthase |
|  | PA0999_pqsD | -6.81 | | -4.86 | | | -16.98 | | 3-oxoacyl-[acyl-carrier-protein] synthase III |
|  | PA1000_pqsE | -6.66 | | -4.62 | | | -15.71 | | Quinolone signal response protein |
|  | PA1001_phnA | -6.52 | | -4.81 | | | -15.81 | | anthranilate synthase component I |
|  | PA1002_phnB | -6.79 | | -5.91 | | | -13.55 | | anthranilate synthase component II |
|  | PA1003_mvfR |  | |  | | |  | | Transcriptional regulator |
|  | PA1216 | -6.20 | | -7.81 | | | -12.51 | | Hypothetical protein |
|  | PA1333 |  | |  | | |  | | Hypothetical protein |
|  | PA1430 |  | |  | | |  | | Hypothetical protein |
|  | PA1431_rsaL |  | |  | | | 1.70 | | Repressor of *lasI* AHL synthase |
|  | PA1456_cheY |  | |  | | | 1.52 | | two component response regulator |
|  | PA1556 |  | | 2.41 | | | 1.87 | | Probable cytochrome C oxidase subunit |
|  | PA1656 |  | |  | | |  | | Hypothetical protein |
|  | PA1673 |  | | 2.54 | | | 2.01 | | Hypothetical protein |
|  | PA1776 |  | |  | | |  | | Probable sigma-70 factor |
|  | PA1914 | -23.43 | | -44.72 | | | -44.02 | | Probable ADP-rybosyl transferase |
|  | PA2031 | -5.62 | | -6.25 | | | -4.77 | | Hypothetical protein |
|  | PA2067 | -3.11 | | -2.71 | | | -3.16 | | Probable hydrolase |
|  | PA2069 | -4.01 | | -2.77 | | | -4.05 | | Probable carbamoyl transferase |
|  | PA2134 | -2.30 | | -2.88 | | | -2.29 | | Hypothetical protein |
|  | PA2146 | -1.97 | | -3.80 | | | -2.62 | | Hypothetical protein |
|  | PA2159 |  | | -3.91 | | | -3.47 | | Hypothetical protein |
|  | PA2172 |  | | -2.66 | | | -2.79 | | Hypothetical protein |
|  | PA2173 |  | | -2.75 | | | -2.43 | | Hypothetical protein |
|  | PA2193_hcnA |  | |  | | | -2.84 | | Hydrogen cyanide synthase |
|  | PA2194_hcnB |  | |  | | | -2.41 | | Hydrogen cyanide synthase |
|  | PA2195_hcnC |  | |  | | | -1.78 | | Hydrogen cyanide synthase |
|  | PA2204 |  | | 2.46 | | |  | | Probable binding protein component of ABC transporter |
|  | PA2274 | -11.4 | | -5.83 | | | -18.23 | | Probable monooxygenase |
|  | PA2299 | -2.13 | | -2.14 | | | -2.30 | | Probable transcriptional regulator |
|  | PA2300_chiC | -11.95 | | -13.11 | | | -8.69 | | chitinase |
|  | PA2486 |  | |  | | |  | | Hypothetical protein |
|  | PA2501 |  | | 2.48 | | | 4.13 | | Hypothetical protein |
|  | PA2570_pa1L | -4,86 | | -5.13 | | | -4.98 | | Galactose-specific PA-I lectin (LecA) |
|  | PA2620_clpA |  | |  | | |  | | ATP-binding protease component |
|  | PA2747 |  | | -1.63 | | | -1.58 | | Hypothetical protein |
|  | PA2753 |  | | 2.96 | | | 2.8 | | Hypothetical protein |
|  | PA2754 |  | |  | | | 2.19 | | Hypothetical protein |
|  | PA2885 |  | | 1.69 | | |  | | Probable transcriptional regulator |
|  | PA3031 |  | |  | | |  | | Hypothetical protein |
|  | PA3126_ibpA |  | | 2.11 | | | 5.03 | | Heat shock protein |
|  | PA3351 |  | |  | | | 2.17 | | Bacterioferritin |
|  | PA3361_lecB |  | | -1.67 | | | -1.51 | | Fucose binding lectin PA-IIL |
|  | PA3369 | -2.11 | | -1.95 | | | -1.97 | | Hypothetical protein |
|  | PA3370 |  | | -2.23 | | | -1.95 | | Hypothetical protein |
|  | PA3371 |  | | -2.4 | | | -2.28 | | Hypothetical protein |
|  | PA3478_rhlB | -3.79 | | -3.16 | | | -1.75 | | Rhamnosyltransferase chain B |
|  | PA3479_rhlA | -2.86 | | -3.01 | | | -1.83 | | Rhamnosyltransferase chain B |
|  | PA3520_copP | -1,91 | | -2.08 | | |  | | Heavy metal associated containing periplasmic metal-binding protein |
|  | PA3531_bfrB |  | | 4.27 | | | 1.86 | | Bacterioferritin |
|  | PA3678_mexL |  | |  | | | 1.73 | | Transcriptional regulator |
|  | PA3691 |  | | -1.69 | | | -1.75 | | Hypothetical protein |
|  | PA3692 | -1,95 | | -2.37 | | | -2.78 | | Probable outer membrane protein precurser |
|  | PA3721_nalC | 2.46 | | 1.73 | | | 4 | | Transcriptional regulator |
|  | PA3812_iscA |  | |  | | |  | | Probable iron binding protein |
|  | PA3813_iscU |  | | -1.54 | | |  | | Probable iron binding protein |
|  | PA3973 | 3.61 | | 2.61 | | | 4.54 | | Probable transcriptional regulator |
|  | PA4078 | -6.28 | | -6.61 | | | -6.41 | | Probable non-ribosomal peptide synthase |
|  | PA4079 | -2.84 | | -2.44 | | | -2.30 | | Probable secreted dehydrogenase |
|  | PA4141 | -4.76 | | -4.52 | | | -2.20 | | Hypothetical protein |
|  | PA4205_mexG | -23.88 | | -10.88 | | | -95.79 | | Hypothetical protein |
|  | PA4206_mexH | -15.51 | | -9.33 | | | -46.71 | | Probable RND efflux membrane fusion protein precurser |
|  | PA4207_mexI | -9.58 | | -7.99 | | | -45.44 | | Probable RND efflux transporter |
|  | PA4208_opmD | -10.08 | | -6.91 | | | -27.83 | | Probable outer membrane protein precurser |
|  | PA4209_phzM |  | | -1.62 | | | -5.09 | | Probable 0-methyltransferase |
|  | Ig 4713795-4713098 |  | |  | | |  | | Intergenic region between *phzA1* and *phzM* |
|  | PA4210_phzA1 | -5.65 | | -4.66 | | | -10.65 | | Probable phenazine biosynthesis protein |
|  | PA4211_phzB1 | -4.13 | | -3.31 | | | -7.57 | | Probable phenazine biosynthesis protein |
|  | PA4212_phzC1 | -3.01 | | -2.84 | | | -8.83 | | Phenazine biosynthesis protein |
|  | PA4213_phzD1 | -4.49 | | -3.46 | | | -10.77 | | Phenazine biosynthesis protein |
|  | PA4214_phzE1 | -3.45 | | -2.97 | | | -9.47 | | Phenazine biosynthesis protein |
|  | PA4215_phzF1 | -3.60 | | -3.17 | | | -10.42 | | Phenazine biosynthesis protein |
|  | PA4216_phzG1 | -3.25 | | -2.56 | | | -7.08 | | Probable pyridoxamine 5’-phosphate oxidase |
|  | PA4217_phzS | -2.34 | | -1.85 | | | -8.39 | | FAD-dependent monooxygenase |
|  | PA4352 |  | |  | | |  | | Hypothetical protein |
|  | PA4387 |  | | 2.52 | | | 4.08 | | Hypothetical protein |
|  | PA4441 |  | |  | | | 2.16 | | Hypothetical protein |
|  | PA4500 |  | |  | | |  | | Probable binding protein component of ABC transporter |
|  | PA4542_clpB | 3.42 | | 2.23 | | | 2.12 | | Heat-shock chaperone |
|  | PA4587_ccpR |  | | 1.67 | | | -1.64 | | Cytochrome *C551* peroxydase precursor |
|  | PA4739 |  | |  | | | -1.89 | | Hypothetical protein |
|  | PA4793 |  | |  | | |  | | Hypothetical protein |
|  | PA4876_osmE | -2.5 | | -2.27 | | | -2.05 | | Osmotically inducible lipoprotein |
|  | PA4877 | -1.77 | | -1.79 | | |  | | Hypothetical protein |
|  | PA4880 |  | |  | | |  | | Probable bacterioferritin |
|  | PA4917 |  | |  | | |  | | Hypothetical protein |
|  | PA4919_pncB1 |  | |  | | |  | | Nicotinate phosphoribosyltransferase |
|  | PA4940 |  | | 1.71 | | | 1.74 | | Hypothetical protein |
|  | PA5015_aceE |  | |  | | |  | | Pyruvate dehydrogenase |
|  | PA5027 |  | |  | | |  | | Hypothetical protein |
|  | PA5053_hslV |  | | 2.73 | | | 4.35 | | Heat-shock protein |
|  | PA5054_hslU |  | | 2.43 | | | 2.01 | | Heat-shock protein |
|  | PA5170_arcD |  | |  | | | 1.80 | | Arginine/ornithine anti-porter |
|  | PA5171_arcA |  | |  | | |  | | arginine deaminase |
|  | PA5212 |  | |  | | |  | | Hypothetical protein |
|  | PA5303 |  | | 2.65 | | | 3.59 | | Hypothetical protein |
|  | PA5461 |  | | 1.57 | | |  | | Hypothetical protein |
|  | PA5481 | -1.84 | | -2.29 | | | -3.29 | | Hypothetical protein |
|  | PA5482 | -2.26 | | -2.04 | | | -3.29 | | Hypothetical protein |
|  | PA5495_thrB |  | | 1.85 | | | 2.89 | | Homoserine kinase |
|  | tRNA-Ser |  | |  | | |  | |  |
|  | tRNA-Cys |  | |  | | | -1.76 | |  |
|  | tRNA-Tyr |  | |  | | |  | |  |
| Total genes | 122^(a)^ | 48 (40%) | | 77 (63%) | | | 88 (73%) | |  |
|  | **Negatively regulated by MvfR (22 genes) ^b)^** | | | | | | | |  |
|  | PA0048 | | -5.01 | | | -3.73 | |  | Probable transcriptional regulator |
|  | PA0049 | | -10.25 | | | -7.17 | | -1.68 | Hypothetical protein |
|  | PA1024 | |  | | |  | |  | Probable electron transfer flavoprotein |
|  | PA1852 | |  | | |  | |  | Hypothetical protein |
|  | PA1984 | |  | | |  | | -2.44 | Probable aldehyde dehydrogenase |
|  | PA2327 | |  | | |  | | -2.08 | Probable permease of ABC transporter |
|  | PA2329 | | -2.30 | | | -1.67 | | -2.91 | Probable ATP-binding component of ABC transporter |
|  | PA2331 | | -2.92 | | | -2.09 | | -3.36 | Hypothetical protein |
|  | PA2662 | |  | | |  | | 1.84 | Hypothetical protein |
|  | PA2663 | | 2.29 | | |  | | 4.04 | Hypothetical protein |
|  | PA3186_oprB | |  | | | -3.59 | |  | Glucose outer membrane porin |
|  | PA3189 | |  | | | -1.62 | | 1.94 | Probable permease of ABC sugar transporter |
|  | PA3195_gapA | |  | | |  | | 1.68 | glyceraldehyde 3-phosphate dehydrogenase |
|  | PA3335 | |  | | | 2.33 | | 2.09 | Hypothetical protein |
|  | PA3392_nosZ | | 4.79 | | |  | | 5.89 | Nitrous-oxide reductase precursor |
|  | PA3403 | |  | | |  | |  | Hypothetical protein |
|  | PA3569_mmsB | |  | | | -2.23 | | -3.5 | 3-hydroxyisobutyrate deshydrogenase |
|  | PA5436 | |  | | | 2.74 | | 2.43 | Probable biotin carboxylase subunit of a transcarboxylae |
|  | Ig (4956028-4956733) | |  | | | -7.46 | |  | Intergenic region between PA4421 and PA4422 |
|  | Ig (727608-721556) | |  | | | -3.42 | |  | Ribosomal RNA cluster between PA0669 and PA0668 (opposite strand) |
|  | tRNA-His | |  | | |  | |  |  |
|  | tRNA-Trp | |  | | | 2.19 | | 1.73 |  |
| Total genes | 22 ^(b)^ | | 6 (27%) | | | 12 (54%) | | 14 (64%) |  |
| **Total number of repressed and activated genes** | | | | | | | | |  |
|  | 144 ^(a+b)^ | | 54 (38%) | | | 89 (62%) | | 102 (71%) |  |

a) Includes the 119 repressed genes from Déziel *et al*. [25], plus *phzH, rhlA* and *phzA1*; b) Includes the 22 activated genes from Déziel *et al*. [25]

Numbers in parenthesis are the percentage of the total MvfR-dependent genes whose expression are altered in response to AA analog treatment.
