# Supplementary material for: Inhibitors of Pathogen Intercellular Signals as Selective Anti-Infective Compounds
Source: PLoS Pathog. 2007 Sep 14;3(9):e126. doi: 10.1371/journal.ppat.0030126 (PMC2323289; doi:10.1371/journal.ppat.0030126)
Supplement: Table S3 — (50 KB DOC) [file ppat.0030126.st003.doc]

**Table S3: Levels of prominent ^1^H HRMAS MR spectra metabolite peaks in PA14 cells minus or plus 4CABA treatment**

| Chemical Shift ^a^  (ppm) | Metabolite | PA14 ^b^  Mean±SD (n=3) | 4CABA ^b^  Mean±SD (n=3) | 4CABA % change from PA14 **^†^** |
| --- | --- | --- | --- | --- |
| 0.94 | Lipids | 0.17±0.04 | 0.32±0.16 | +91 |
| 1.92 | Acetate | 0.101±0.019 | 0.16±0.06 | +58 |
| 3.05 | Lysine | 0.130±0.003 | 0.14±0.03 | +11 |
| 3.21 | Choline | 0.041±0.004 | 0.065±0.03 | +60 |
| 3.27 | Betaine Aldehyde | 0.11±0.04 | 0.23±0.05 | +117 *(*P*=0.029) |
| 3.57 | Glycine | 0.069±0.011 | 0.10±0.010 | +51 *(*P*=0.015) |
| 3.92 | Betaine Aldehyde | 0.084±0.027 | 0.20±0.04 | +142 *(*P*=0.013) |
| 5.99 | C/UXP | 0.0110±0.0017 | 0.021±0.004 | +86 *(*P*=0.017) |
| 6.14 | ADP+ATP | 0.0204±0.020 | 0.030±0.003 | +47 ***** (*P*=0.011) |
| 7.96 | C/UXP | 0.0095±0.012 | 0.014±0.0016 | +51 *(*P*=0.014) |
| 8.19 | NAD | 0.0014±0.002 | 0.0265±0.0013 | +95 *(*P*=0.001) |
| 8.27 | ADP+ATP | 0.027±0.003 | 0.031±0.005 | +16 |
| 8.43 | NAD | 0.012±0.005 | 0.020±0.003 | +73 |
| 8.84 | NAD | 0.010±0.004 | 0.0132±0.0018 | +36 |
| 9.15 | NAD | 0.009±0.003 | 0.0107±0.0013 | +21 |
| 9.34 | NAD | 0.007±0.004 | 0.0117±0.0015 | +63 |

^a^, chemical shifts according to Bundy *et* *al*. [51]; and Chauton *et* *al*. [52].

^b^, ratio calculated from the height of the metabolite to the height of the reference (TSP).

*, denotes statistical significance, *P* ≤ 0.05; independent-samples t-test (equal variances, two-tailed; α=0.05). The null hypothesis of equal variances failed to be rejected (omnibus ANOVA test, *P*=0.470; and Levene’s test; SPSS 12.0, SPSS, Inc.) for each and all n*=*3 metabolite peak measurements. Assumption of normality was tested for each of the n*=*3 metabolite peak measurements (Shapiro-Wilk test, R Language). For the 4CABA-treated cells, the Shapiro-Wilk test indicated violation of normality at 3.21 ppm (*P=*0.02), but a Mann-Whitney U-test between the 3.21 ppm metabolite values from PA14 and 4CABA-treated bacteria resulted in no significant difference (*P*=0.100, exact, two-tailed, SPSS 12.0). In all other measurement sets, assumption of normality failed to be rejected with *P*-values ranging from 0.08 to 0.99 (Shapiro-Wilk *P*-values).

^†^Calculated as 100x [(4CABA peak height - PA14 peak height)/PA14 peak height].
